# Soil indigenous microbiome and plant genotypes cooperatively modify soybean rhizosphere microbiome assembly

**DOI:** 10.1186/s12866-019-1572-x

**Published:** 2019-09-02

**Authors:** Fang Liu, Tarek Hewezi, Sarah L. Lebeis, Vince Pantalone, Parwinder S. Grewal, Margaret E. Staton

**Affiliations:** 1 Department of Entomology and Plant Pathology, University of Tennessee, 153 Plant Biotechnology Building, 2505 E.J. Chapman Drive, Knoxville, TN 37996 USA; 2 Department of Plant Science, University of Tennessee, 252 Ellington Plant Sciences Building, 2431 Joe Johnson Drive, Knoxville, TN 37996 USA; 3 Department of Microbiology, University of Tennessee, 513 Ken and Blaire Mossman Bldg, 1311 Cumberland Avenue, Knoxville, TN 37996 USA; 4 Department of Plant Science, University of Tennessee, 254 Plant Biotechnology Building, 2505 E.J. Chapman Drive, Knoxville, TN 37996 USA; 5 College of Science, University of Texas Rio Grande Valley, 1201 W. University Drive, Edinburg, TX 78539 USA; 6 Department of Entomology and Plant Pathology, University of Tennessee, 154 Plant Biotechnology Building, 2505 E.J. Chapman Drive, Knoxville, TN 37996 USA

**Keywords:** Rhizosphere, Microbiome, Soybean genotypes, Microbe-microbe interactions, Plant-microbe network

## Abstract

**Background:**

Plants have evolved intimate interactions with soil microbes for a range of beneficial functions including nutrient acquisition, pathogen resistance and stress tolerance. Further understanding of this system is a promising way to advance sustainable agriculture by exploiting the versatile benefits offered by the plant microbiome. The rhizosphere is the interface between plant and soil, and functions as the first step of plant defense and root microbiome recruitment. It features a specialized microbial community, intensive microbe-plant and microbe-microbe interactions, and complex signal communication. To decipher the rhizosphere microbiome assembly of soybean (*Glycine max*), we comprehensively characterized the soybean rhizosphere microbial community using 16S rRNA gene sequencing and evaluated the structuring influence from both host genotype and soil source.

**Results:**

Comparison of the soybean rhizosphere to bulk soil revealed significantly different microbiome composition, microbe-microbe interactions and metabolic capacity. Soil type and soybean genotype cooperatively modulated microbiome assembly with soil type predominantly shaping rhizosphere microbiome assembly while host genotype slightly tuned this recruitment process. The undomesticated progenitor species, *Glycine soja*, had higher rhizosphere diversity in both soil types tested in comparison to the domesticated soybean genotypes. *Rhizobium*, *Novosphingobium*, *Phenylobacterium*, *Streptomyces*, *Nocardioides,* etc. were robustly enriched in soybean rhizosphere irrespective of the soil tested. Co-occurrence network analysis revealed dominant soil type effects and genotype specific preferences for key microbe-microbe interactions. Functional prediction results demonstrated converged metabolic capacity in the soybean rhizosphere between soil types and among genotypes, with pathways related to xenobiotic degradation, plant-microbe interactions and nutrient transport being greatly enriched in the rhizosphere.

**Conclusion:**

This comprehensive comparison of the soybean microbiome between soil types and genotypes expands our understanding of rhizosphere microbe assembly in general and provides foundational information for soybean as a legume crop for this assembly process. The cooperative modulating role of the soil type and host genotype emphasizes the importance of integrated consideration of soil condition and plant genetic variability for future development and application of synthetic microbiomes. Additionally, the detection of the tuning role by soybean genotype in rhizosphere microbiome assembly provides a promising way for future breeding programs to integrate host traits participating in beneficial microbiota assembly.

**Electronic supplementary material:**

The online version of this article (10.1186/s12866-019-1572-x) contains supplementary material, which is available to authorized users.

## Background

It has been widely recognized that plants utilize associated microbes for a range of beneficial functions including nutrient acquisition, pathogen resistance and stress tolerance [1]. Recent studies consistently demonstrate that the plant microbiome greatly extends plants’ adaptations to changing environments [2, 3]. These results suggest a promising new avenue of research for sustainable agriculture [4]. Further, microbe community assembly is not static or passive; plants can actively modulate the assembly of their beneficial microbiome in response to stressors (e.g., drought and pathogen infection). This dynamic response further highlights the possibility of optimizing crop yields by exploiting beneficial plant-microbe interactions [2, 5, 6].

The rhizosphere is an interface between plant root and soil characterized by a dynamic microbial community with intensive microbe-microbe and plant-microbe communication mediated by plant molecular signals, especially secondary metabolites [7]. At this root-microbe interface, plant and microbes have evolved intimate interactions. Plants allocate a significant portion of photosynthates as root exudates that serve as resources for microbes, and in return, microbes help to increase plant fitness via various plant growth promoting impacts [4, 8]. The rhizosphere is also the first line of plant defense to pathogen infection [1] and acts as the initial filter for the subset of microbes that will colonize the root as endophytes [9]. Understanding the major factors that shape the rhizosphere microbiome assembly and the mechanisms of mutual adaptation between microbes and plants in response to changing environmental conditions will help to identify potential targets for future crop breeding and management.

Comprehensive characterization and comparison of rhizosphere microbiomes among numerous plant species under different conditions has consistently revealed the crucial impacts of soil source [9, 10] and plant genetic traits [11–13] on rhizosphere microbiome assembly. The pool of microbes available in the soil determines the initial microbial repertoire for this assembly process [7]. In addition, soil physio-chemical characteristics directly modulate microbial communities and may also indirectly alter rhizosphere microbiome assembly through impacts on host plant physiology [7]. Plant physiology and genetics also control rhizosphere composition. Differences in root morphology and in the quantity and quality of rhizodeposits could greatly diversify the composition and activity of the rhizosphere microbiome in a species-specific way [7]. With the advantage of nitrogen fixation by rhizobia, the root exudates of legumes differs from non-legumes in both quantity and quality, with higher exudation amounts and lower carbon-to-nitrogen ratios [14]. This special trait of legumes may shape rhizosphere microbiome assembly differently compared with non-legume plant. Turner et al. (2013) compared rhizosphere microbiomes between wheat, oat, and pea and found a higher rhizosphere effect (i.e, compositional and functional difference of microbiome between rhizosphere and nearby soil) in pea compared with the cereals. In addition to soil source and plant genetic traits, domestication, soil nutrient status and abiotic stress mediate rhizosphere microbiome assembly to different degrees [11, 15–17].

The impact of plant genotypes on rhizosphere microbiome composition is usually reported to be very weak but varies depending on soil context and plant species studied [18]. For example, composition of the rice root microbiome was significantly influenced by rice genotype when grown under controlled greenhouse conditions, whereas no impact was detected under field conditions [19]. Peiffer et al. (2013) suggested a small but significant impact of maize genetic variations on bacterial diversity under field conditions by a comprehensive comparison across 27 inbred lines. A comparison of the rhizosphere microbiome between barley genotypes with different domestication histories also revealed small but significant impacts, and these genotype-dependent impacts were manifested by differing the abundance of a few specific taxa instead of whole community-level differences [15]. Although genotype level modification of microbial composition appears to be modest, genes participating in immune response, nutrient response, and stress response could change the abundance of specific microbial consortia, which in turn would profoundly alter host performance [16, 17, 20, 21]. One example of this change was reported by Hanley et al. (2015), in which genotype differences in the ability to associate with *Pseudomonas fluorescens* between wild *Arabidopsis* accessions were found to be related to host fitness [22].

Soybean is an important crop worldwide as an essential food resource for protein and vegetable oil and also is the largest feedstock source for biodiesel production in the United States [23–26]. Soybeans form a symbiotic relationship with the nitrogen-fixing rhizobia. As improvement of nitrogen-fixing capacity of soybeans is a major research goal, numerous studies have been conducted to understand the process and signaling pathways that mediated this symbiotic interaction. Soil physico-chemical characteristics, including soil moisture, temperature, pH and nutrient status, have consistently been reported as crucial factors determining the efficiency of nodulation and nitrogen fixation [27–29]. Due to this predominant symbiotic interaction between rhizobia and soybean, the microbiome composition of soybean may differ from non-legume plants. This difference was observed in the root microbiome of another legume, *Trifolium pratense,* in which rhizobia accounted for 70% of the whole root microbiome [30].

To evaluate the relative contribution of soil source and host genetic traits in rhizosphere microbiome assembly, six soybean genotypes with varying traits and two soil types with distinct microbiome compositions were chosen to compare rhizosphere microbiome assembly both compositionally and functionally. Considering the distinct developmental traits of the genotypes and distinguished microbiome difference between soil types, we hypothesize that both factors will significantly and cooperatively manipulate the structure and composition of rhizospheric microbiota. It has been recognized that microbe-microbe interaction is another crucial driving force for rhizosphere microbiome assembly [15, 31]. To examine this factor, we also compared the difference of microbial network patterns between bulk soil and rhizosphere and among genotypes in terms of the network complexity, modularities, and key taxa. By integrating the information from differential abundance analysis, microbial network, and metabolic pathway results, we aim to establish a foundation of knowledge about how the soybean rhizosphere is structured.

## Results

A total of 19,358,039 raw reads from 136 samples were generated after paired-end sequencing with a read length of 275 bp. Quality analysis with FastQC suggested that the first 200–250 bp of each read had a quality score higher than 30 (Additional file 1: Figure S1), and 88–95% of sequences had an exact match in the primer region. After several steps of stringent trimming and filtering of chimeric and non-bacterial sequences, 9,945,986 reads were clustered into 175,957 OTUs based on a threshold of 97% sequence similarity. Most of the samples yielded about 50,000 reads, with the minimum sequencing depth of 19,023 and the maximum depth of 247,930 (Additional file 1: Figure S2). The rarefaction curve suggested consistent bacterial OTU richness across samples, with no obvious outlier samples (Additional file 1: Figure S3). After rarefaction to the minimum sequencing depth, 76,864 OTUs remained in the 136 samples, belonging to 25 phyla, 99 classes, 122 orders, 244 families and 642 genera.

### Soybean rhizosphere demonstrates different but dependent microbial community composition compared to bulk soil

Overall, the microbial community of the soybean rhizosphere microbiome is significantly different from that of bulk soil, with some taxa being consistently recruited to the rhizosphere regardless of the soil type. However, some other bacterial taxa were specifically enriched in soybean rhizosphere in a soil-dependent way.

#### Phylum, class, order and family level comparison

**Fig. 1:**
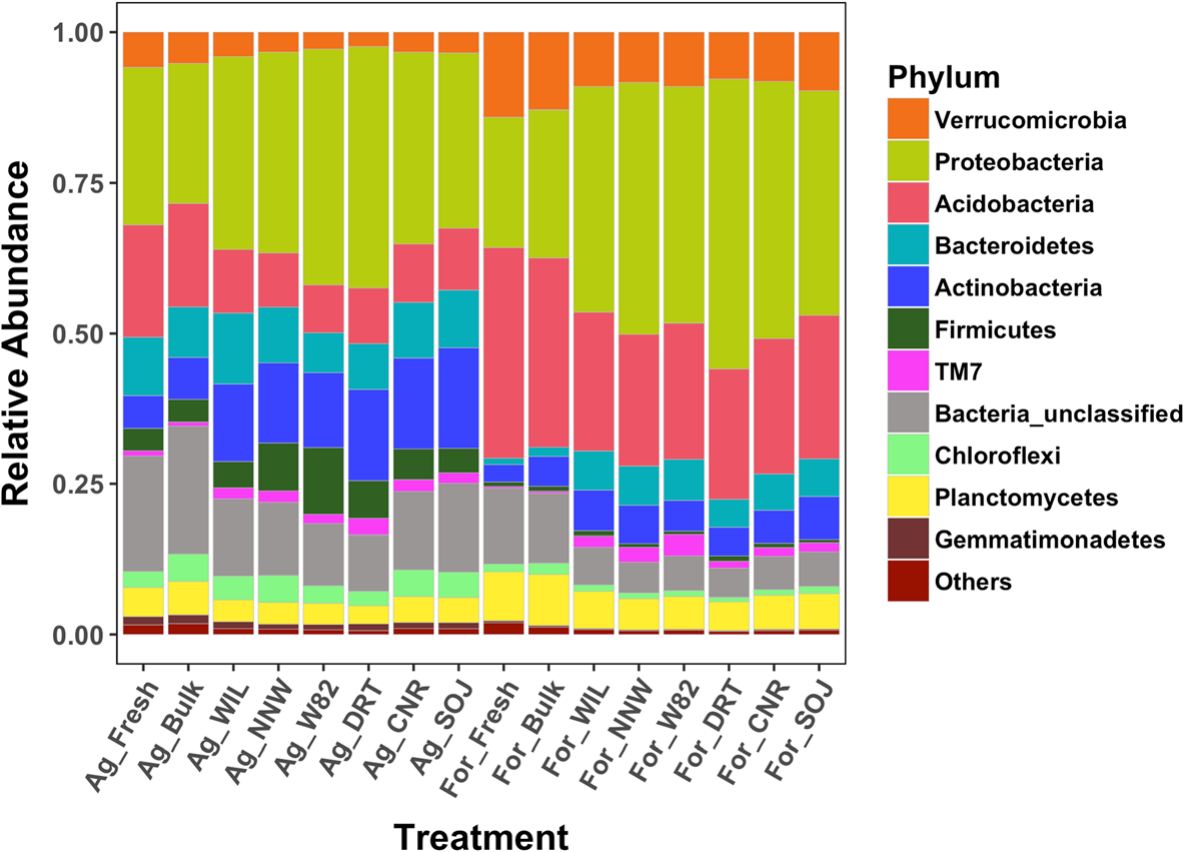
Bacterial community composition at phylum level. Bacterial phyla with relative abundance smaller than 1% across 20% of samples were grouped together to form the “Others” category. Fresh soil was soil sample flash frozen immediately after field collection, while bulk soil was those treated the same as rhizosphere but without soybean grown in it

**Fig. 2:**
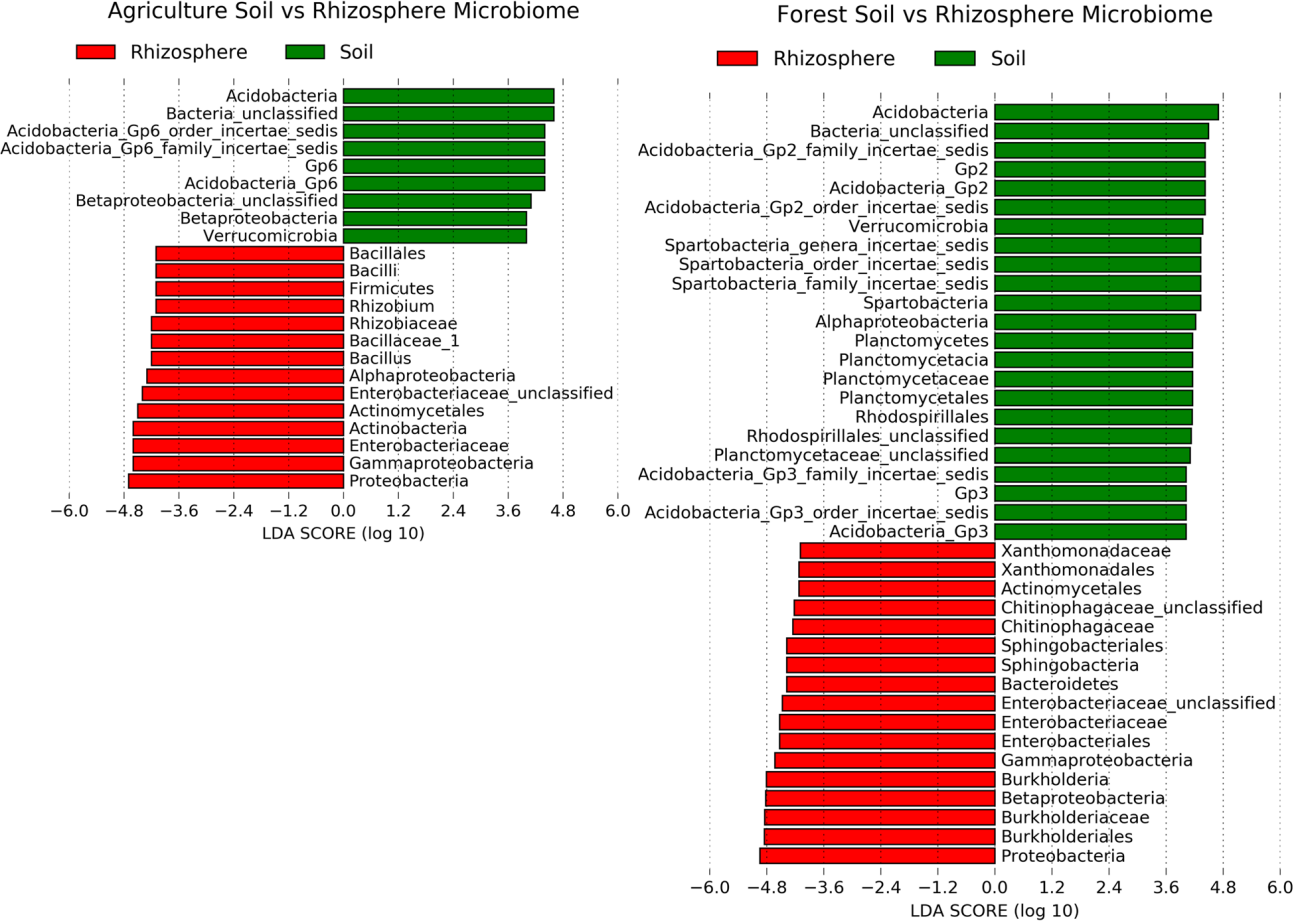
Differential abundance between soil and rhizosphere by linear discriminant analysis (LDA) > 4. In this LefSe analysis, soil samples (including both fresh and bulk samples) were treated as controls. A negative LDA score represents depletion in soil and enrichment in rhizosphere (red) and a positive LDA score represents the opposite (green)

At the phylum level, bacterial communities were dominated by *Proteobacteria*, *Acidobacteria*, *Actinobacteria,* and *Bacteroidetes* in both agricultural and forest soils, with the next most abundant phyla being *Firmicutes* in agriculture soil, and *Verrucomicrobia* and *Planctomycetes* in forest soil (Fig. 1). The composition of microbes immediately after collection (fresh soil) and after 2 months in the greenhouse (bulk soil) were similar, indicating that the greenhouse environment and the time lapse did not largely alter microbial communities. Comparison of bulk and fresh soil samples to rhizosphere samples revealed much greater differences. Differential abundance analysis results indicated that *Proteobacteria*, *Actinomycetales* and *Enterobacteriaceae* were significantly enriched from bulk soil to rhizosphere in both soil types across all the six genotypes, while *Acidobacteria* and *Verrucomicrobia* were consistently depleted in soybean rhizosphere (Fig. 2). However, the enrichment/depletion pattern of bacterial phyla in the soybean rhizosphere was not entirely consistent between soil types; *Firmicutes* (especially *Bacilli*) was preferably enriched in the rhizosphere when grown in agriculture soil, while *Bacteroidetes* (specifically *Chitinophagaceae*) were selectively accumulated when growing in forest soil. Similarly, *Alphaproteobacteria* (especially *Rhizobiales*) and *Betaproteobacteria* (specifically *Burkholderiales*) were discriminately enriched in agriculture and forest soil respectively. Although Gammaproteobacteria was consistently enriched in the rhizosphere across all treatments, the enrichment of bacteria within the Gammaproteobacteria class differed between soil types, with *Xanthomonadaceae* preferably enriched in forest soil while *Pseudomonadaceae* were preferably recruited when grown in agriculture soil. This divergent enrichment/depletion pattern in soybean rhizosphere between soil types indicates the dominant impacts of the soil sources and their starting microbial pools on rhizosphere microbiome assembly.

#### Genus level

**Fig. 3:**
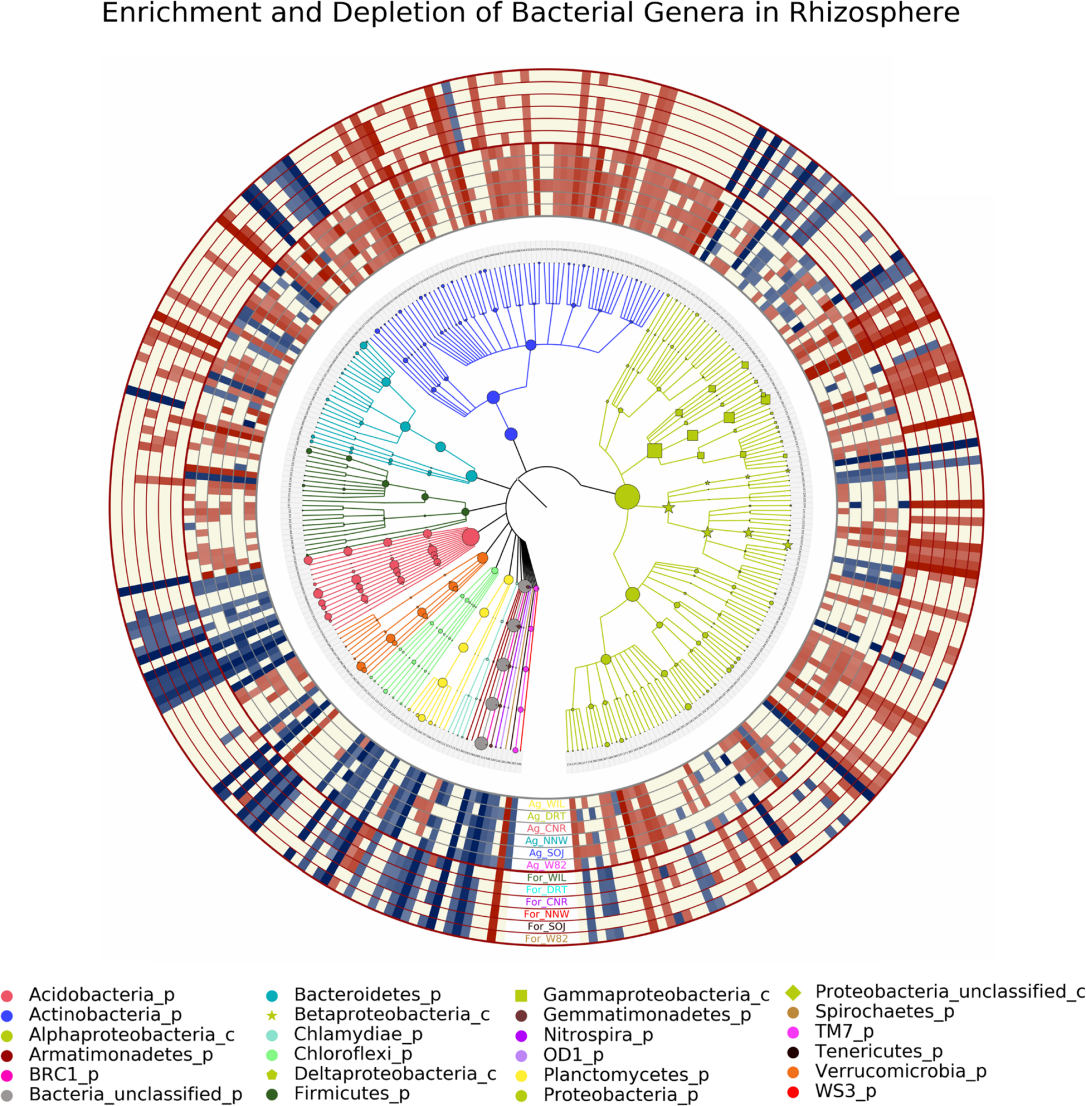
The enrichment and depletion of bacteria by genera in the soybean rhizosphere. The inside dendrogram represents the taxonomic tree of all bacterial genera with significantly different abundance between soil and rhizosphere, with color indicating phylum. Proteobacteria (green) were subset to class level, with circle, star, pentagon, square and diamond representing Alpha-, Beta-, Delta-, Gamma- and Unclassified- Proteobacteria respectively. The number at the end of each branch represents the corresponding bacterial genus as annotated along the list along each side of the plot. A detailed annotation list could be found in Additional file 2. The enrichment/depletion of each genus in the soybean rhizosphere is depicted in the external heatmap ring, with red indicating enrichment, blue representing depletion, and yellow indicating no significant difference. The darker the color of each block, the stronger the corresponding enrichment/depletion, which is scaled based on corresponding LDA score

To provide more detailed understanding of bacteria assembly in soybean rhizosphere under different soil conditions and host genetic background, *LefSe* analysis was conducted at the genus level to determine the enrichment/depletion pattern between each pair of rhizosphere and soil samples (e.g., Ag_WIL rhizosphere vs. soil samples) with an LDA score threshold of 2. In total, the relative abundances of 299 out of 642 bacterial genera were detected to be significantly different between rhizosphere and soil samples. Among these 299 genera, 11 were consistently enriched in the soybean rhizosphere for both soil types across the six genotypes: *Rhizobium*, *Novosphingobium*, *Phenylobacterium*, *Streptomyces*, *Nocardioides*, *Nocardia*, *Amycolatopsis*, *Dyadobacter*, *TM7_genus_incertae_sedis*, *Sphingobacteriaceae_unclassified*, and *Enterobacteriaceae_unclassified*. In contrast, 11 out of the 299 genera (*Gp15*, *Gp13*, *Gp9*, *Gp6*, *Gemmata*, *Rhodospirillales-unclassified*, *Betaproteobacteria-unclassified*, *Rhodocyclaceae-unclassified*, *Deltaproteobacteria-unclassified*, *Planctomycetaceae-unclassified*, and *Bacteria-unclassified*) were steadily depleted in the rhizosphere (Fig. 3).

**Fig. 4:**
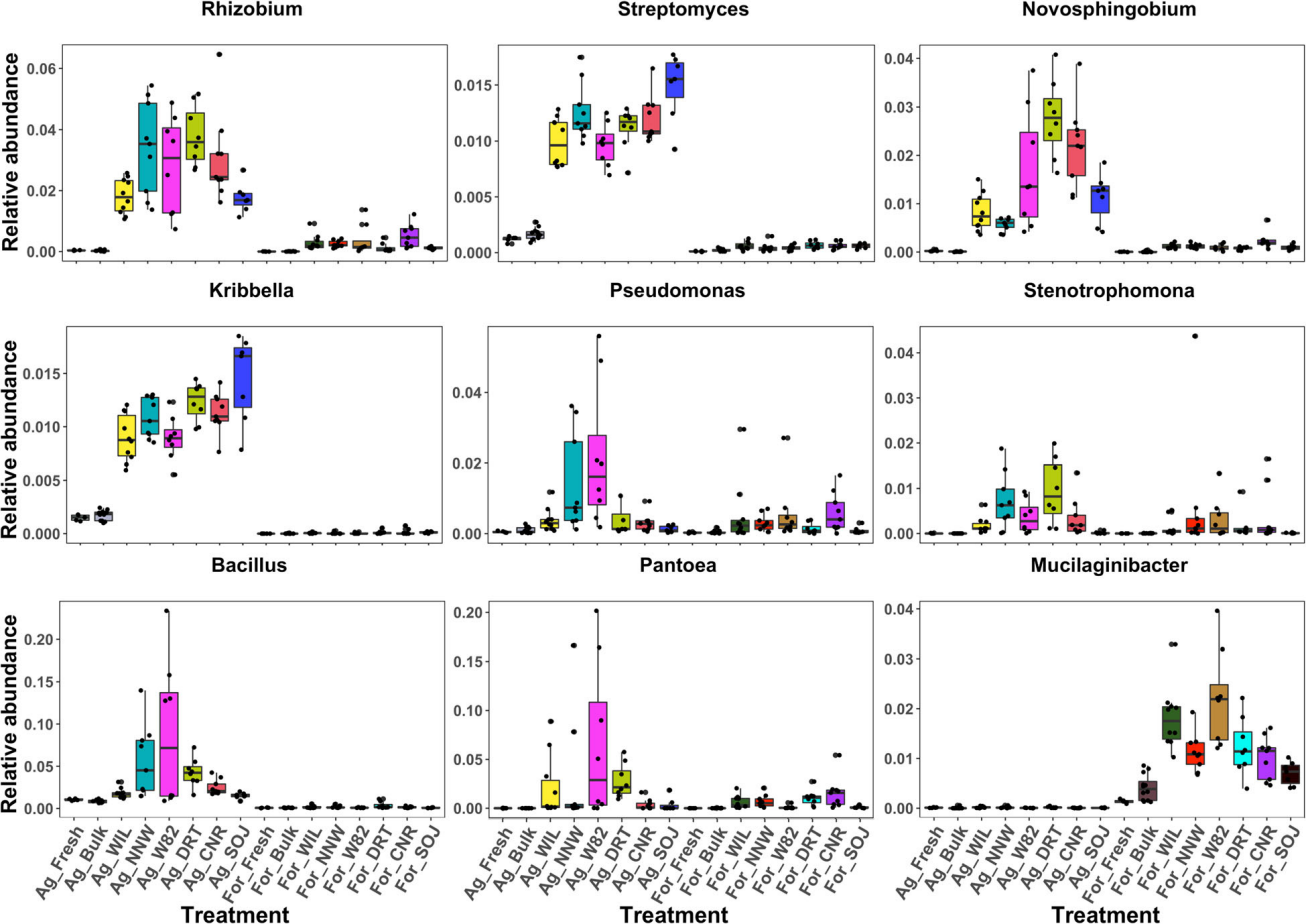
Boxplot of bacterial genus abundance between treatments

Consistent with phylum level results, numerous bacterial genera were selectively enriched/depleted in the rhizosphere when grown in one soil type instead of the other. For example, *Bradyrhizobium*, *Pseudoxanthomonas*, *Kribbella*, *Agromyces*, etc. were favorably accumulated in the soybean rhizosphere when grown in agriculture soil. Meanwhile, *Burkholderia*, *Rudaea*, *Dyella* and *Mucilaginibacter*, etc. were discriminatively recruited to the soybean rhizosphere when grown in forest soil. Likewise, *Gp1* and *Pasteruria* were significantly decreased in the soybean rhizosphere when grown in agriculture soil while *Gp2* was selectively depleted when grown in forest soil. In total, 37 genera were specifically enriched in the rhizosphere when soybeans were grown in agriculture soil while 13 genera were specifically enriched in forest soil (Additional file 3). Among the 37 specifically enriched genera, only one genus was absent in the soybean rhizosphere when growing in forest soil, while none out of the 13 genera was absent in soybean rhizosphere when grown in agriculture soil. In other words, this soil-type specific bacteria enrichment may be attributed to differences in rhizosphere assembly processes instead of absence of a specific taxon in the microbial pool. Additionally, even among those that were consistently enriched, the degree of the enrichment also varied and depended on the soil type. For example, differential abundance analysis indicated that *Rhizobium*, *Streptomyces* and *Novosphingobium* were constantly enriched in soybean rhizosphere across all genotypes and soil types. However, the degree of this enrichment was more dominant when grown in agriculture soil compared with that of forest soil (Fig. 4). In contrast, the depletion of *Acidobacteria* was more distinct in soybean rhizosphere when the plants were grown in forest soil in comparison with those grown in agriculture soil.

In addition to soil type effects, between-genotype differences in bacteria enrichment/depletion patterns were also apparent (Fig. 3). As visualized in the bacterial genus abundance boxplots, *Pseudomonas* and *Stenotrophomonas* were enriched in all soybean genotypes except *Glycine soja* (Fig. 4). Similarly, the recruitment of *Rhizobium*, *Pantoea* and *Mucilaginibacter* in *Glycine soja* was also limited compared with the other five genotypes. However, the recruitment of *Streptomyces* and *Kribbella* was more evident in the wild species accession (SOJ) compared with other genotypes when grown in agricultural soil. Compared with other genotypes, non-nodulating soybeans (NND) were less attractive to *Novosphingobium* as demonstrated by its lower abundance in soybean rhizosphere.

### Dominant impacts of soil indigenous microbe pool and soil environment on rhizosphere microbial community composition

**Fig. 5:**
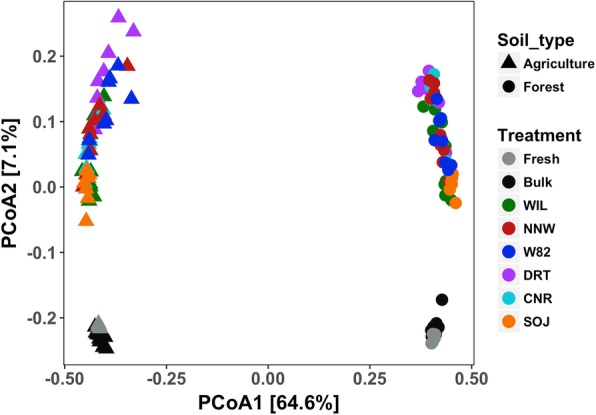
Bacterial community composition between treatment. Agriculture and forest soil types were represented by triangle and circle correspondingly. Different colors of the points represent different treatments

To quantify the differences in microbial community composition between samples, Bray-Curtis dissimilarity was calculated and visualized in a PCoA plot. The separation pattern between samples indicated distinct microbial community composition between the rhizosphere and bulk soil as well as between soil types (Fig. 5). The first two axes explained more than 70% of microbial community variance between samples, with samples clearly separated by soil type on the first axis (64.6% explained variance), while compartment (rhizosphere or bulk soil) was primarily represented along the second axis (7.1% explained variance).

To evaluate the relative impacts of soil type and compartment on microbial community composition, a PERMANOVA was used to partition the source of variance. Here, the compartment impacts were referred to microbiome differences between soil samples and soybean rhizosphere samples. The results suggested that soil type is the most dominant explanatory factor for the distinct microbial community structure between samples, explaining 62% of the overall variance of the microbe composition across all samples (PERMANOVA marginal effects: *F*_*(1,131)*_ = 259.65, *p* < 0.001). Larger soil type effects for bulk and fresh soil microbial communities (81.37% variance) were detected compared with the rhizosphere microbiome (70.61%). Compartment effects were the secondary key factor (explaining 6% of variance) that contributed to the overall divergent microbial community (PERMANOVA marginal effects: *F*_*(2,131)*_ = 12.86, *p* < 0.001). The compartment effects are in fact mainly contributed by the rhizosphere, considering the very similar microbiome composition between fresh and bulk soil samples. Thus, compartment effects will be referred to as rhizosphere effects hereafter. Within each individual soil type, the rhizosphere effects were more evident, with 28.16% (PEMANOVA marginal effects: *F*_*(2,65)*_ = 12.74, *p* < 0.001) and 38.48% (PERMANOVA marginal effects: *F*_*(2,65)*_ = 20.33, *p* < 0.001) variance of microbiome composition being explained in agriculture and forest soil correspondingly. A significant interaction of soil type and rhizosphere effects was also detected for the overall microbiome composition (PERMANOVA marginal effects: *F*_*(2,129)*_ = 12.67, *p* < 0.001). The impact of sequencing depth on microbe composition results was evaluated and found to be nonsignificant when soil type and compartment were taken into account altogether (PERMANOVA marginal effects: *F*_*(1,131)*_ = 1.815, *p* = 0.138).

### Soybean genotype slightly tunes soybean rhizosphere microbiome assembly

**Fig. 6:**
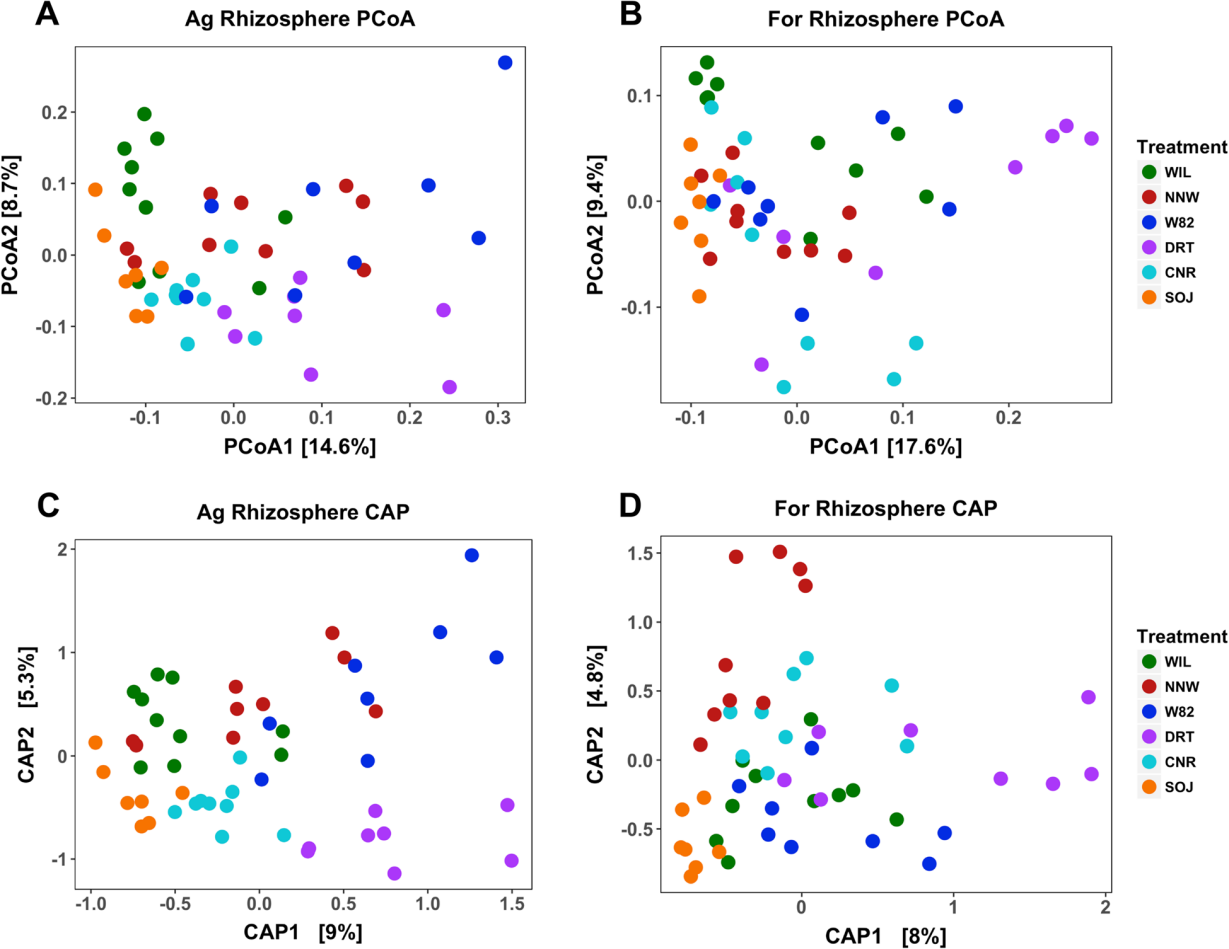
Genotype impacts on soybean rhizosphere microbiome assembly. Principal coordinate analysis (PCoA) plot of soybean rhizosphere microbial community grown in agriculture soil (**a**) and forest soil (**b**) demonstrated little pattern associated with genotype. In contrast, soybean rhizosphere microbial community difference between genotypes as depicted by canonical analysis of principal coordinates (CAP) has more clear genotype-specific patterns, with **c** and **d** representing rhizosphere samples grown in agriculture and forest soil, respectively

To evaluate the impacts of soybean genotype on rhizosphere microbiota assembly, the dataset was subdivided into two subsets composed of agriculture and forest rhizosphere samples. A PERMANOVA test indicated significant impacts of the soybean genotype in both agriculture (PERMANOVA marginal effects, *F*_*(5,45)*_ = 2.70, *p* < 0.01) and forest (PERMANOVA marginal effects, *F*_*(5,45)*_ = 2.44, *p* < 0.01) rhizosphere microbe composition, with 23.08 and 21.32% variance explained respectively. The differences driven by genotypes were not evident when visualized using an unconstrained ordination method, i.e., PCoA (Fig. 6a and b). However, when illustrated using canonical analysis of principal coordinates (CAP), the influence of microbe community compositions due to genotypes is more clear (Fig. 6c and d). CAP analysis is a good option when effects are not easily detected by unconstrained ordination, as it can utilize treatment information [32]. Genotype impacts were more evident for soybeans grown in agriculture soil, with the drought-resistant genotype (DRT) and wild-type genotype (SOJ) more divergent from others (Fig. 6c and d). In contrast, the bacterial community structure of Williams (WIL), Williams non-nodulating mutant (NNW) and Williams 82 (W82), all of which share the Williams genetic background, were more similar and had no clear separation pattern on the CAP plot. Significant interactive impacts of soil type and genotype were detected in determining soybean rhizosphere microbiome composition (PERMANOVA marginal effects: *F*_*(5,89)*_ = 2.03, *p* = 0.04).

Another important aspect of variability worth examining is flowering time. All rhizosphere samples were taken as soon as plants reached the flowering stage, in order to mitigate the impact of different developmental stage that might impact the results. However, as the six genotypes are from different maturity groups (i.e. are adapted to different climatic zones), the individual soybeans in this study flowered at different times over the course of 6 weeks. A PERMANOVA test suggested significant impacts of flowering time on both agriculture and forest rhizosphere microbe composition. After partialling out flowering time as a factor, the soybean genotype still explained 3% of the variance (capscale, *F*_*(1,39)*_ = 2.29, *p* < 0.01). Due to the high correlation between flowering time and genotypes, it is difficult to rule out the pure genotype effects on rhizosphere microbiome assembly from that of flowering time when tested using all samples. To help evaluate the soybean genotype impacts, we grouped samples that flowered on the same date and visualized their rhizosphere microbiome composition with a PCoA plot (Fig. S4). We observed distinct rhizosphere microbiome composition between Williams (WIL) and the non-nodulating mutant of Williams (NNW). These two genotypes are genetically identical other than a mutation of gene *R*_*j5,6*_, which is a receptor gene of rhizobia nodulation factor [33]. The divergent rhizosphere microbiomes between these two genotypes indicate that their genetic difference indeed confers direct impact on rhizosphere composition independent of flowering time differences.

### Significant rhizosphere effects on microbiome diversity and microbe-microbe interactions

**Fig. 7:**
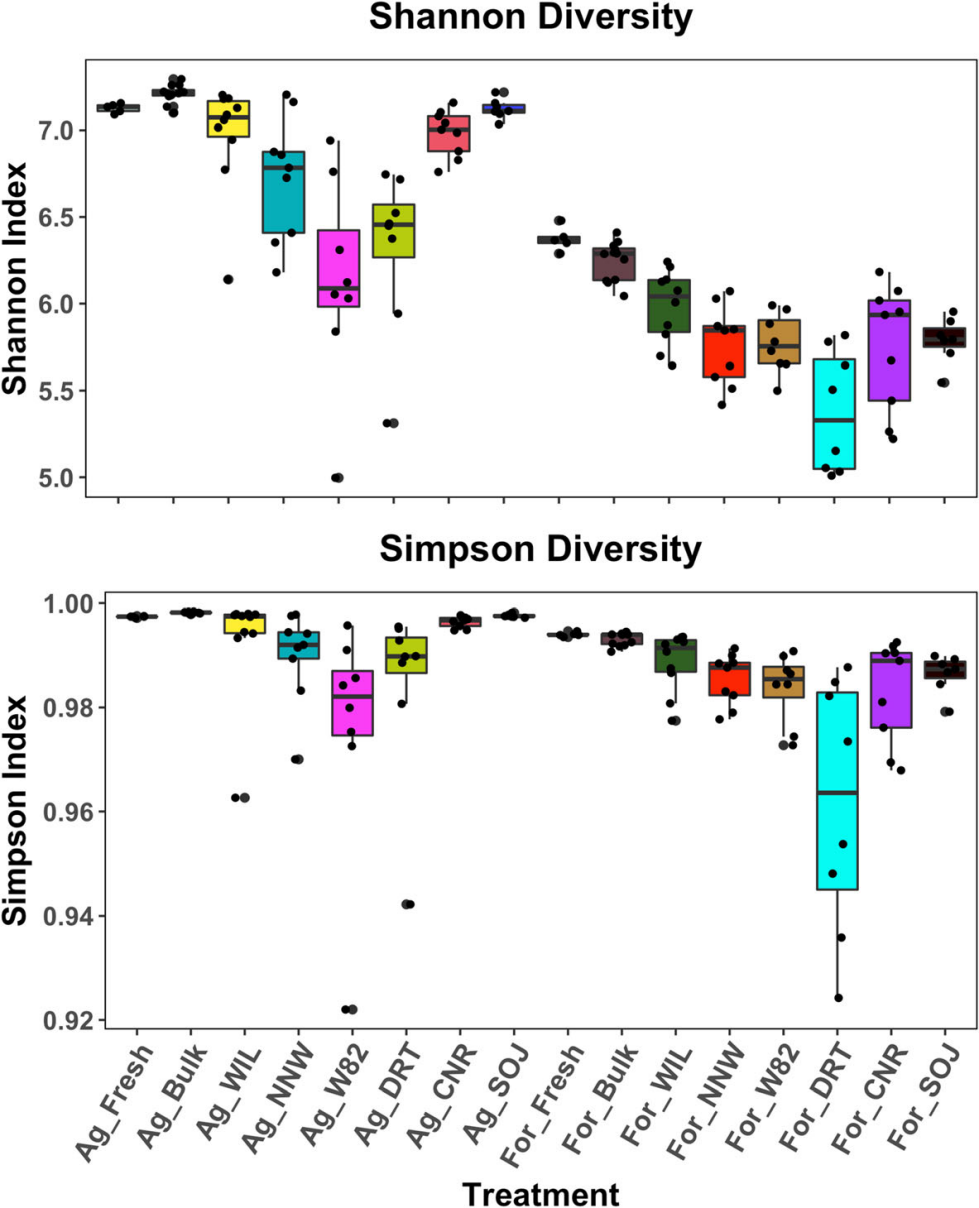
Rhizosphere effects on microbiome diversity

Indigenous microbial community diversity was significantly higher in agriculture soil than forest soil, which held true for both bulk soil and the soybean rhizosphere (*F*_*(1,130)*_ = 228.82, *p* < 2.20e-16) (Fig. 7). A significant rhizosphere effect was reflected by reduced microbiome diversity in soybean rhizosphere compared with that of fresh and bulk soil samples (*F*_*(2,130)*_ = 23.96, *p* = 1.39e-09), with no significant difference detected between the latter two. Rhizosphere microbiome diversity also differed significantly between genotypes in both agriculture (ANOVA; *F*_*(5,45)*_ = 9.46, *p* = 3.22e-06) and forest soil (ANOVA; *F*_*(5,45*_ =4.99, *p* = 0.10e-02). The diversity of the drought-tolerant genotype (DRT) was significantly and consistently smaller than other genotypes in both soil types. In addition, there was a significant interaction effect of soil type and genotypes on rhizosphere microbiome diversity (*F*_*(5,90)*_ = 4.42, *p* = 0.12e-02).

Beyond the direct modulation by soil and plant host, the interactions between microbes act as another selective force for root microbiome assembly [31]. To elucidate these interactions in the rhizosphere and infer key microbial consortia, we characterized co-occurrence correlation networks between microbes and compared the difference of those interaction patterns between treatments. Overall, microbe-microbe interactions in soil were more dense and connected compared with that of rhizosphere as indicated by higher edge density and average connection degree in soil samples (Table 1), which is consistent with the reduced bacterial diversity in the rhizosphere. The complexity of the microbial network in WIL was consistently higher than the other five genotypes in both soil types. However, there were no strong correlations between network complexity and microbial diversity when fitted using linear regression between average network density and Shannon diversity (Additional file 1: Figure S5).

**Table 1:** Network topological characteristics. Global statistics were calculated based on co-occurrence network comprising all significant microbe-microbe correlations at α < 0.001 while top 50 statistics were calculated based on simplified network that including only the top 50 nodes with the most connections to other microbes

Soil type	Network type	Network topologies	Soil	WIL	DRT	CNR	NNW	SOJ	W82
Agriculture	Global	Positive edges^a^ (%)	54.64	56.69	58.74	57.33	57.68	55.91	57.77
		Edge density^b^ (%)	2.35	1.58	0.76	0.81	0.64	0.93	0.73
		Ave. degree^c^	25.68	16.83	8.46	8.93	6.97	9.32	6.11
		Betweenness^d^	1092.03	1294.41	1588.35	1576.23	1691.40	1471.82	1405.33
	Top 50	Positive edges^a^ (%)	48.45	50.66	93.28	48.75	62.26	66.67	90.91
		Edge density^b^ (%)	76.49	49.47	11.88	13.61	6.79	15.54	8.42
		Ave. degree^c^	37.48	24.24	5.58	6.53	2.65	7.15	4.04
		Betweenness^d^	5.76	12.38	34.02	34.73	29.20	34.47	46.34
Forest	Global	Positive edges^a^ (%)	56.25	55.68	56.21	55.42	57.60	54.81	55.70
		Edge density^b^ (%)	1.49	0.88	0.73	0.68	0.77	0.75	0.74
		Ave. degree^c^	16.77	9.75	8.38	7.43	8.75	8.54	8.59
		Ave. Betweenness^d^	1273.30	1541.86	1653.03	1626.60	1611.71	1589.82	1626.15
	Top 50	Positive edges^a^ (%)	58.03	60.66	70.33	94.57	84.72	45.54	77.27
		Edge density^b^ (%)	63.02	24.90	7.74	10.69	19.61	9.76	6.67
		Ave. degree^c^	30.88	12.20	3.71	4.38	8.83	4.39	2.93
		Ave. Betweenness^d^	9.06	23.30	55.98	56.24	27.04	47.76	62.22

^a^Positive ratio represents the ratio of positive microbe-microbe correlations out of all interactions within the network

^b^Edge density was calculated as the ratio of detected edge numbers to the theoretical maximum edge numbers, indicating the connectiveness between nodes

^c^Ave. degree was defined as the mean connection degree across all nodes within a network

^d^Ave. Betweenness was defined by the average number of shortest paths going through all vertices within a network

When all of the significant microbe-microbe interactions were taken into account at 훼< 0.01, there was no significant separation of the rhizosphere microbiome networks between soybean genotypes, but the difference between soil types was distinguishable (Additional file 1: Figure S6). The connection degree of each node varied between 1 and 337, with the top 25 most connected OTUs belonging to *Mycobacterium*, *Sphingomonas*, *Massilia*, *Bradyrhizobium*, *Bacillus*, *Gp16*, *Streptomyces*, *Phenylobacterium*, *Rhizobium* and *TM_genus_incertae_sedis* genera. A high percentage of nodes were shared between soil and rhizosphere networks, with 64–72% of nodes being shared in the two compartments in agriculture soil, while 71–75% overlap between compartments was detected in forest soil. The positive correlation ratios (the positive microbe-microbe correlations out of all significant interactions) were detected to be higher in the soybean rhizosphere compared with soil samples. To evaluate the correlation of taxa abundance and its connection densities, linear regression models were fitted using OTU relative abundance and corresponding node degree (Additional file 1 Figure S7). The results showed weak but significant correlation between OTU abundance and corresponding node degree. Several OTUs with high abundance showed limited interactions with other taxa, including OTU000004 and OTU000012, belonging to *Burkholderia* and *Rhizobium* respectively. In contrast, several rare taxa such as OTU000159 and OTU000349, belonging to *Mycobacterium* and *Spartobacteria_genera_incertae_sedis* showed high degree of connections with other bacteria.

To simplify the network and identify key microbe-microbe interactions, the top 50 OTUs with the highest connection degrees were selected from each treatment for detailed comparison. Within this subset, the network complexity of soil samples was still consistently higher than that of rhizosphere (Table 1). The network of WIL was denser compared with other genotypes in both soil types. However, the network pattern of the other five genotypes, such as network density and positive correlation ratio, varied between soil types (Additional file 1: Figure S8). When grown in agricultural soil, DRT, SOJ and W82 had higher positive interactions than other genotypes whereas CNR, NNW and W82 had with higher positive interactions when growing in forest soils. These results again confirm the cooperative modulating role of soybean genotypes and indigenous soil types in microbe-microbe interactions.

**Fig. 8:**
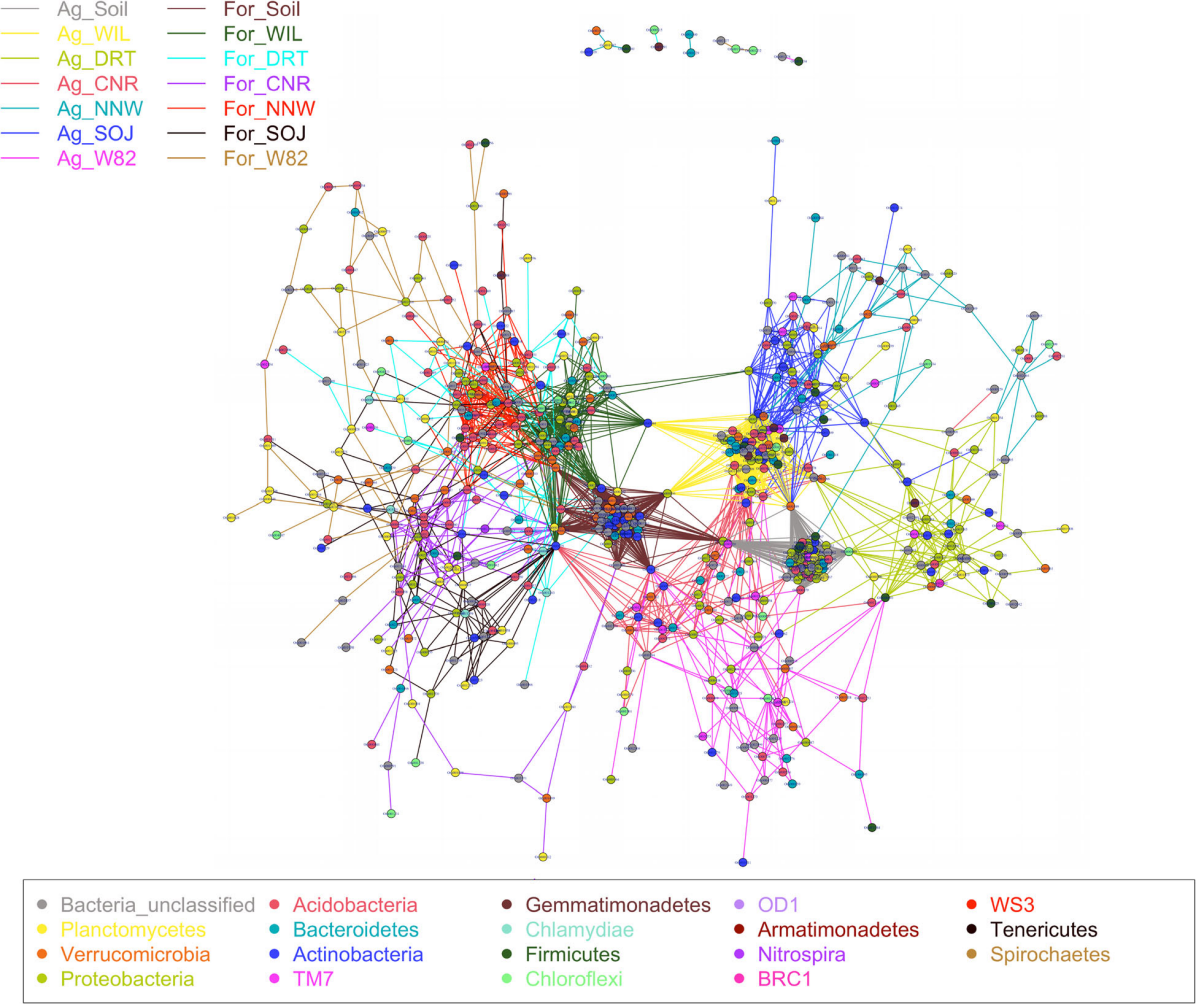
Modulation of microbial networks by soybean genotypes and soil source. In the above network, OTUs were represented by individual nodes, with colors indicating phylum. Edge color denotes the treatment. When one edge was shared between treatment, a mixed color was used to define that particular edge. OTU numbers are labeled for each node and their corresponding taxonomic information can be found in Additional file 4

To understand the overall network patterns between treatments, the individual top 50 networks were united to a comprehensive network based on shared OTUs between treatments (Fig. 8). After the union process, the number of nodes was reduced from 700 to 566, with most belonging to *Proteobacteria* (105), *Bacteria_unclassified* (95), *Acidobacteria* (91), *Planctomycetes* (55), *Actinobacteria* (54), *Verrucomicrobia* (51) and *Bacteroidetes* (47). OTUs with the highest number of connections with others belonged to *Bradyrhizobium*, *Mycobacterium*, *Sphingomonas*, *Gp4*, *Spartobacteria_genera_incertae_sedis*, *TM7_genus_incertae_sedis*, *Massilia* and *Gp16*. The differences in microbe-microbe interactions between soil types and among genotypes were exemplified by the high modularity of subnetworks between soybean genotypes, which was strikingly different than the analysis that included all significant correlations. In contrast to the large percentage of shared OTUs between treatments when all significant OTUs were taken into account, only a few OTUs were shared between soil and rhizosphere as well as among genotypes when the top 50 key microbes were concerned. These shared OTUs function as connectors between the subnetworks (Fig. 8) and are classified in the genera *Bacillus*, *Streptomyces*, *Bradyrhizobium*, *Rhizobiales_unclassified*, *Arthorobacter*, *Caldilineal*, *Mycobacterium*, and *Gp1* as well as several unclassified genera in the phylum of *Verrucomicrobia*. Such bacterial consortia may play a dominant and persistent role in modulating microbial community composition via prevalent interactions with other bacteria.

### Specialized microbiome function in soybean rhizosphere

**Fig. 9:**
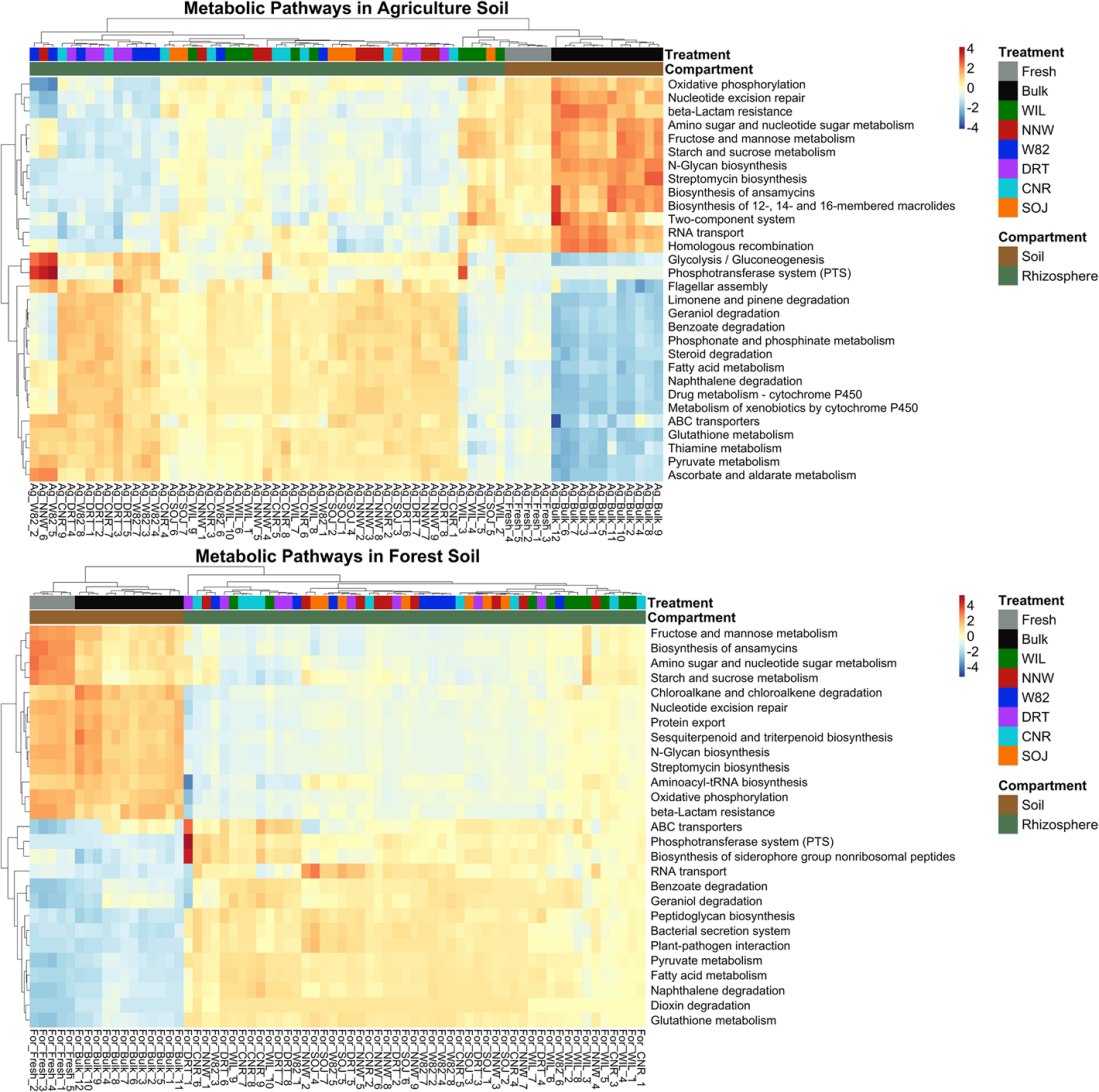
Rhizosphere effects on soybean microbiome metabolic capacity. Metabolic pathways that differed significantly between soil and rhizosphere were used to generate a heatmap. Both samples and pathways were clustered based on Euclidean distance. The abundance of each pathway was scaled to the same range (− 4, 4), with red and blue colors representing relatively higher and lower abundance respectively

Genotype-specific rhizosphere effects were detected in the soybean rhizosphere microbiome as reflected by differential microbial community compositions between rhizosphere and bulk soil as well as among genotypes. To understand the functional differences of these communities, we predicted the potential metabolic capacities of both the soil and rhizosphere microbiomes using *Tax4Fun*. The results indicated divergent metabolic capacities between soybean rhizosphere microbiota and bulk soil community (Fig. 9). Of particular interest, the enrichment/depletion of metabolic pathways was consistent between soil types and across genotypes regardless of the divergent bacteria composition.

Metabolic pathways related to biodegradation of xenobiotics, including glutathione metabolism, geraniol degradation, limonene and pinene degradation as well as naphthalene degradation, were significantly and consistently enriched in the soybean rhizosphere regardless of soil types. Pathways involved in nutrient transformation and transport, such as phosphotransferase systems and ABC transporters were also enriched in soybean rhizosphere. In addition, bacterial functions related to plant-microbe interactions were also enriched in the rhizosphere, such as flagella assembly, bacterial secretion system, and biosynthesis of siderophore. In contrast, metabolic pathways involved in antibiotic production, including streptomycin biosynthesis and biosynthesis of ansamycins, were enriched in the bulk soil environment. The metabolic pathways for fructose, mannose, starch and sucrose metabolism were accumulated in soil as well. Another functional group significantly expanded in soil bacteria involved DNA repair and recombination including nucleotide excision repair and homologous recombination.

## Discussion

### Soil type-dependent rhizosphere effects

In our study, *Proteobacteria*, *Acidobacteria*, *Bacteroidetes*, and *Actinobacteria* were the most dominant bacterial phyla in soybean rhizosphere, which is consistent with previous reports about the soybean rhizosphere microbiome [34–36]. *Gammaproteobacteria* and *Actinomycetales* were consistently enriched in the soybean rhizosphere in both soil types, which is consistent with the thought that *Actinobacteria* and *Proteobacteria* as copiotrophs are more competitive in a nutrient-enriched environment like rhizosphere, while oligotrophs like *Acidobacteria* and *Verrucomicrobia* are more abundant in soil with poor nutrients [18]. However, at the genus level, this enrichment exhibited difference for some specific bacteria genera within *Gammaproteobacteria* and *Actinomycetales*, which greatly depended on soil types and soybean genotypes. This result indicates that analysis based on different taxonomic levels may achieve inconsistent conclusions about the robustness of rhizosphere bacteria assembly. Considering the functional redundancy between different bacteria, functional analysis of rhizosphere microbiome together with compositional characterization maybe more informative for understanding microbiome assembly and promoting applications for sustainable agriculture.

Rhizosphere effects on bacteria composition have been widely recognized on numerous plant species, such as maize [10], rice [19], *Arabidopsis thaliana* [9], alfalfa [36], poplar [37], grapevine [38], and sugarcane [39]. These investigations spanned monocotyledons and dicotyledons, annuals and perennials, and legumes and non-legumes. The results found differing extents of rhizosphere effects between plant species due to distinct evolution time, plant root physiology and root exudation profile between species [11, 40]. Turner et al. (2013) revealed a stronger rhizosphere effect of microbial community on pea (legume) compared with that of oat and wheat [13]. Similarly, *Lotus japonicus* plants assemble a distinct rhizosphere microbial community that is influenced by root nodule symbiosis [41]. In our study, strong rhizosphere effects were validated in soybean as reflected by the distinct microbial community composition and structure between rhizosphere and bulk soil. These rhizosphere effects may be influenced by the specific profile of root exudates with a high concentration of flavonoids, which are essential components of signal exchange between soybean and symbiotic rhizobia during nodule formation. The influence of root exudates was also investigated by While et al. (2015), revealing that isoflavonoids also significantly alter soybean rhizosphere bacterial diversity [42].

In our study, a number of well-described plant growth promoting rhizobacteria (PGPR) [43], including *Rhizobium*, *Dyadobacter*, *Novosphingobium* and *Streptomyces*, were consistently enriched in soybean rhizosphere. PGPR greatly expanded host adaptations and performance by various promoting activities, including IAA and siderophore production, phosphate solubilization, and induced systemic resistance [43, 44]. Strong enrichment of *Streptomyces* and *Dyadobacter* was also detected in the rhizosphere of pea [13]. A diverse of *Rhizobium* colonize soybean root and form nodules, providing significant benefits to the plant through nitrogen fixation. The enrichment of *Rhizobium* in the soybean rhizosphere, even in the non-nodulating soybean variety, corroborates the idea that rhizosphere recruitment may be an important first step for further selection to the rhizoplane and endosphere [45], which facilitates symbiotic interactions between bacteria and host plants.

Despite the similarities in soybean rhizosphere microbe assembly across soil types, discriminant enrichment between soil types was also revealed in our study, reflecting the dominant modulating role of the indigenous microbe pool and local soil conditions. *Bradyrhizobium*, *Kribbella*, and *Agromyces* were selectively enriched in the soybean rhizosphere when the plants were grown in agricultural soil with a neutral pH and diverse bacteria pool. In contrast, *Burkholderia* and *Mucilaginibacter* were discriminatively accumulated in the soybean rhizosphere grown in forest soil with an acidic pH and less diverse bacterial pool. *Burkholderia* has been found to be enriched near roots grown under extremely nutrient-deficient soil and function to metabolize organic acid exuded by the host to soluble phosphate [46, 47]. This result is consistent with the selective enrichment of *Burkholderia* in forest soil with lower pH. Recent research confirms that dynamic root exudates from plants can interact with microbial substrate preference to shape the rhizosphere microbiome community composition [48], providing a promising avenue of research to understand the underlying mechanisms driving this selective enrichment process. Despite the predominant dependency of the soybean rhizosphere microbiome assembly on soil type, we found that the impacts of soil types on rhizosphere microbe composition was smaller in comparison to corresponding impacts on indigenous soil microbial community. This result indicates that soybean as a plant host intrinsically exerts some conserved modulating force in shaping the rhizosphere microbiome assembly. In addition, rhizosphere effects were exhibited to a higher degree when soybeans were grown in forest soil compared to those grown in agricultural soil, indicating that the degree of the rhizosphere effect differs depending on the environment. This may indicate that the plants exert variable influence on the rhizosphere microbiome depending on the environment, possibly in response to how suitable the environment is for the plant growth.

### Soybean genotypes mildly tuning rhizosphere microbiome assembly

The modulating role of plant genotypes to rhizosphere microbiome assembly is thought to be much smaller in modern agriculture systems and domesticated crops compared with that of natural systems with a long history of coevolution [4, 7]. In our study, moderate and significant tuning effects by soybean genotypes on the rhizosphere microbiome composition were detected from both the overall bacterial community level and the individual genus level. At the community level, the rhizosphere microbiome composition from Williams (WIL), Williams 82 (W82) and Williams non-nodulating mutant (NNW) were more similar, while drought resistant and wild type plants were more distinct. This corresponds to the genetic differences among the genotypes, with the Williams (WIL), Williams 82 (W82) and Williams non-nodulating mutant (NNW) all sharing the Williams genetic background. Intriguingly, this between-genotype difference was detected to be more evident when soybean genotypes were grown in agriculture soil. This soil type-dependent genotype effects again indicates the integrated regulatory role from both the soil and the plant side.

Previous work in various agricultural plant species has revealed domestication to be a profound shaping force for rhizosphere microbiome recruitment, influenced by both the reduced genetic diversity of modern genotypes and crop management practices [18]. Several studies revealed distinct microbial community composition in wild genotypes compared with that of modern genotypes [15, 49–51]. This study also found the rhizosphere bacteria community composition of the wild accession - *Glycine soja* (SOJ) - to be different from the other modern agricultural genotypes. Specifically, the enrichment of *Rhizobium*, *Pseudomonas* and *Stenotrophomonas* in the wild soybean rhizosphere was very limited compared with the other modern genotypes. In contrast, *Streptomyces* and *Kribbella* from the *Actinobacteria* phylum were extensively recruited in the wild type. In addition, the overall bacterial diversity in the wild soybean (SOJ) rhizosphere was consistently higher in comparison to all genotypes irrespective of the soil type tested. Similarly, the study by Zachow et al. (2014) revealed that wild sugar beet harbors higher bacteria diversity in its rhizosphere compared with wild type. The distinct rhizosphere microbiome recruitment of the wild accession could be a reflection of soybean trait selection along domestication. For example, root morphology changed significantly from the wild progenitor to the modern agricultural genotypes, with shallow and thick roots being preferably selected during soybean breeding history in terms of phosphorus efficiency [52].

Soybeans benefit from a nitrogen supply provided by the nitrogen-fixing process from the symbiotic relationship with *Rhizobium* and *Bradyrhizobium* that results in a higher quality of root exudates with a lower C/N ratio [14]. Additionally, the nitrogen fixing process alters soil physicochemical properties around root nodules, featuring a high concentration of hydrogen as a by-product of nitrogen reduction by nitrogenase [53]. Considering these specific traits conferred by the nitrogen fixing process, it is reasonable to expect that the bacterial community of non-nodulating genotype (NNW) would significantly differ from its nodulating isogenic line (WIL). However, no effects were detected in our study. The non-nodulating Williams mutant (NNW) selected for this study was established by silencing the *R*_*j5,6*_ gene coding for GmNFR5α and GmNFR5β (*Glycine max* Nod factor receptors), which are orthologs of NFR5 receptor in *Lotus japonicus* [33]. As a result, this mutant exhibited neither rhizobia infection nor cortical cell division. This contrasts with previous research in *Lotus japonicus*, which found that disruption of the symbiosis pathway significantly altered rhizosphere microbial communities, even with the addition of supplemental nitrogen to soil [41]. These contrasting results warrant further investigation, with possible causes including the particular genes selected to disrupt nodulation, different soil nitrogen status, or specific physiologies of the two different plant species.

### Specialized network in rhizosphere and genotype specific preference for key microbe-microbe interactions

As a result of discriminant selection occurring in the soybean rhizosphere, the diversity of the bacterial community in rhizosphere was significantly lower than that of soil. Consistently, microbe-microbe interactions represented by co-occurrence networks were revealed to be less complicated in the rhizosphere compared to soil, which is consistent with previous studies using shotgun metagenomics [34]. We found that high abundance of a bacterial taxa is not necessarily required to be a key species in terms of microbe-microbe interactions. Rare bacteria of *Mycobacterium* were found to have a high number of interactions with other taxa, which may indicate that some rare but essential species play critical roles for community structure through dense connections with other groups [34]. Bacterial taxa that are consistently and highly connected with other groups potentially play key role in community structure and crucial ecological functions [54]. The microbiome network identified in this study could help guide future investigations of plant-microbe interactions by focusing on hub taxa that are highly connected with other groups as well as connector taxa that provide links between modules [55]. When represented using all significant correlations, the microbial networks were quite similar between rhizosphere and soil community as well as among different genotypes. However, after reduction of network complexity by selecting the top 50 taxa, we found that soybean-genotype-featured unique subnetworks were linked together by crucial connector taxa belonging to *Bacillus*, *Mycobacterium*, *Streptomyces* and *Arthrobacter*. This contrasting pattern may indicate that the global microbe-microbe interactions within the complex bacterial community are similar between soybean genotypes, but the key microbe-microbe interactions are genotype-specific.

### Consistent rhizosphere effects on bacterial metabolic capacities between soil types and genotypes

Functional pathway analysis revealed distinct microbial metabolic capacities in the soybean rhizosphere, and these rhizosphere effects were consistent between different soil types and soybean genotypes. Specifically, bacterial functional pathways related to plant-microbe interactions, biodegradation of xenobiotics, as well as nutrient transformation and transport were significantly enriched in the soybean rhizosphere, while antibiotic biosynthesis, DNA repair and recombination related pathways were reduced. Many of the enriched pathways in the rhizosphere have previously been reported to be essential for the various plant growth promoting functions across several studies [15, 56, 57]. For example, flagellar assembly, siderophores and bacterial secretion system were revealed to be involved in induced systemic resistance [43]. Despite the clear influence of the soil type and soybean genotype on bacterial community composition and microbe-microbe interactions in the soybean rhizosphere, our study identified much overlap in the metabolic capacities of the bacterial communities. This convergence may be due to the functional redundancy of various taxa in the bacterial community [58]. However, this study is limited to inferring functional annotation based on taxonomic classification, and further confirmation of actual rhizosphere microbiome functions is warranted.

Plants are not able to escape from unfavorable conditions, such as being attacked by herbivores or pathogens, due to their sessile nature. During their evolution, plants have developed various strategies to directly or indirectly respond to external stressors by exuding various defense compounds into the rhizosphere for instance [59]. To adapt with this specialized habitat, the rhizosphere microbiome may have evolved with increased detoxification activity as reflected by the enhanced degradation pathway of limonene, pinene and naphthalene in our results. This finding is consistent with a former report about the intensive expression of genes involved in oxidative stress response and detoxification in the corn rhizosphere [56]. Our functional characterization of the soybean rhizosphere also showed that common carbon metabolism pathways including starch, sucrose, fructose and mannose metabolism were downregulated. As Boris and Jörg stated that most bacteria are characterized with flexible and dynamic carbon-utilization strategy in response to available carbon sources [60]. This decrease in common carbon metabolism pathway could reflect the adaptation of rhizosphere microbiome to the abundant specialized nutrients being supplied by root exudates. This is consistent with the reports of the special carbon utilization capacities of several plant growth promoting bacteria. For example, *Pseudomonas fluorescens* can use α-pinene as its sole carbon and energy source. Similarly, naphthalene can be utilized as the sole carbon and energy source by several bacterial genera including *Burkholderia*, *Mycobacterium*, *Streptomyces*, *Sphingomonas*, *Pseudomonas*, *Ralstonia* etc. [61, 62]. Surprisingly, we found antibiotic activity to be reduced in rhizosphere, which contradicts previous reports that antibiotic activity of PGPR in rhizosphere are particularly important especially when plants were infected by pathogens [1, 43, 63]. This difference could be due to the different soil nutrient conditions or lack of pathogen stress in our experiment.

## Conclusion

In this study, we provide a detailed characterization of soybean rhizosphere microbiome composition and functional capacity across a number of soybean genotypes and a wild accession. The rhizosphere microbiome composition and microbe-microbe interactions between soybean genotypes and soil types advances our understanding of the modulating role of both factors in the soybean rhizosphere microbiome assembly. This base knowledge primes further studies to use candidate bacteria consortia for synthetic community-based in vitro testing of this assembly process and the functional roles of the bacteria. Our results emphasize the importance of comprehensive consideration of native microbe pool, local soil environment and plant genotypes for future microbiome study. Additionally, the significant genotype tuning role in the soybean rhizosphere microbiome assembly indicates that agricultural breeding programs will need to consider integrating host traits participating in beneficial microbiota assembly.

## Methods

In this study, five soybean genotypes with unique ecological or physiological traits were selected to evaluate genotype impacts on rhizosphere microbiome assembly (Table 2), including cv. Williams (WIL), a drought-tolerant cultivar (DRT), a cyst nematode-resistant line (CNR), a non-nodulating mutant of Williams (NNW), and cv. Williams 82 (W82). An accession of the undomesticated progenitor species of soybeans, *Glycine soja* (SOJ), was also included. The seeds were provided by the USDA, Agricultural Research Service, Germplasm Resources Information Network (GRIN). All soybean seeds were surface sterilized with a 10% sodium hypochlorite solution for 30 mins, followed by three rinses with deionized distilled water. Seeds were germinated on paper in a 26 °C incubator in darkness for 2 days. Germinated soybean seeds were transplanted to autoclaved vermiculite. Just before the soybeans reached trifoliolate stage (about 11 days after germination), fresh agriculture soil of pH around 7.5 was collected from a depth of 20 cm from the East Tennessee AgResearch and Education Center Plant Science Unit. Fresh forest soil was obtained from the University of Tennessee Plateau Research and Education Center, with a soil pH of about 4.8. After field collection, all fresh soils were transported to the greenhouse the same day after collection. After removal of roots and debris, soil was homogenized by mixing, then allocated to pots (diameter = 20 cm). The second day after soil collection, soybean seedlings at the trifoliolate stage were transplanted into the fresh soil and grown in the greenhouse until flowering stage (30 °C day/20 °C night, 16 h light/8 h dark, relative humidity of 60–80%). Fifteen pots of soil without soybeans were used as bulk soil control. Each treatment group (genotype by soil) was started with 10 biological replicates. Both soybean seedlings and control pots were watered as needed every other day.

**Table 2:** Soybean genotype inventories and specific characters

Genotype Abbr.	Plant Inventory	Maturity Group	Cultivar or distinguishing character
WIL	548,631	III	Williams
DRT	416,937	VI	Drought-tolerant with different root morphology
CNR	TN09–029	IV	Soybean cyst nematode-resistant
NNW	634,765	III	Non-nodulating mutant of Williams
SOJ	407,305	V	*Glycine soja* undomesticated progenitor
W82	518,671	III	Williams 82

At the flowering stage, soybean rhizosphere soil samples were collected according to Lundberg et al. (2012). Briefly, the root ball of soybeans were gently removed from the pot and soil loosely attached to the roots was removed by mild shaking. Soybean roots with tightly attached soil were put into a 50-mL centrifuge tube filled with 30 mL of autoclaved phosphate buffer (per liter: 6.33 g of NaH_2_PO_4_.H2O, 16.5 g of Na_2_HPO_4_.7H2O, 200 μL Silwet L-77). The tube was vortexed at maximum speed for 30 s and the slurry was filtered through a 100-μm cell strainer into a new 50-mL centrifuge tube. The soil slurry was then centrifuged to precipitate soil particles. After another round of resuspension and centrifuging, the soil pellet was collected into 1.5 mL eppendorf tubes. To eliminate the interference of the soil crust on microbiome characterization, the surface soil was removed from the control pot and the remaining soil was well homogenized. A similar amount of soil as that of rhizosphere was collected from the soil mix and defined as bulk soil. All of the extracted soil samples were flash frozen in liquid nitrogen and stored at − 80 °C before DNA extraction.

Soil DNA was extracted with the *MoBio* soil DNA extraction kit following the manufacturer’s protocol. Most of the samples yielded concentrations of about 200 ng/μL. 16S rRNA gene based bacteria profiling were accomplished with MiSeq 275 bp paired-end sequencing targeted V3-V4 regions, with forward primer 341F = 5′-CCTACGGGNGGCWGCAG-3′ and reverse primer 785R = 5′-GACTACHVGGGTATCTAATCC-3′ [64]. Library preparation followed the Illumina 16S metagenomic sequencing protocol. Briefly, for the first step PCR, 16S rRNA gene specific primer with adapter overhangs was used to amplify template out of genomic DNA utilizing 2X KAPA HiFi HotStart ReadyMix with the following PCR cycle: 95 °C for 3 min; 25 cycles of 95 °C for 30 s, 55 °C for 30 s, 72 °C for 30 s; 72 °C for 5 min, then hold at 4 °C. During the second step of PCR, dual indices and Illumina sequencing adapters were attached to the template amplified from step one using the Nextera XT Index Kit with PCR cycle: 95 °C for 3 min; 8 cycles of 95 °C for 30 s, 55 °C for 30 s, 72 °C for 30 s; 72 °C for 5 min and hold at 4 °C. To eliminate the amplification of chloroplast and mitochondria sequences from any plant contamination, peptide nucleic acid (PNA), including anti-mitochondrial PNA (mPNA) 5′-GGCAAGTGTTCTTCGGA-3′ and the anti-plastid PNA (pPNA) 5′-GGCTCAACCCTGGACAG-3′ were used to block their elongation during the first step of PCR [65].

*Mothur* software was used to process 16S rRNA gene sequences, including quality control, assembly, alignment, chimera removal, *SILVA*-based OTU clustering at 97% similarity, and naive Bayesian classifier-based OTU classification against Ribosomal Database Project (RDP) training set [66]. During this process, any sequence pairs that have a mismatch within the primer region were removed before assembly. Chimera sequences were detected and removed using the mothur-incorporated vsearch tool based on the UCHIME algorithm [67, 68]. Sequences that belong to chloroplast, mitochondria, eukaryotes, and archaea were discarded before OTU clustering. To alleviate the bias introduced by uneven sequencing depth, rarefaction at the minimum sample sequencing depth (19023) was used for normalization before subsequent microbial community analysis in R.

Beta diversity between samples was calculated with the Bray-Curtis weighted distance, and principal coordinate analysis (PCoA) using this dissimilarity matrix were applied to visualize the differences between microbial communities between treatments. Permutational multivariate analysis of variance (PERMANOVA) was used to evaluate the marginal effects contributed by each factor to the distinct microbial composition pattern between treatments using 999 permutations. In addition to PERMANOVA, partial canonical analysis of principal coordinates (CAP) [69] based on Bray-Curtis distance was used to further evaluate the impacts of genotypes on rhizosphere microbiome assembly and visualized through a CAP plot. Considering the strong similarity of bacterial composition between fresh soil samples (before greenhouse experiment) and bulk samples (after greenhouse experiment), subsequent *LefSe*, network and KEGG pathway analysis were performed on combined bulk soil and fresh soil samples (hereafter were represented as soil treatment).

Differential abundance analysis of bacteria at different taxa levels between treatments were performed with *LefSe* under one-against-all mode (i.e., one taxa is considered to be significantly different only when it is significantly different against all remaining treatments) [70]. The LDA logarithmic score was calculated with 200 bootstraps iterations, and any taxa with α less than 0.05 were defined to be significantly different between treatments. For overall abundance comparison between soil and rhizosphere across all bacterial taxa levels, the LDA logarithmic score threshold was set to 4.0. To provide a comprehensive comparison of bacteria enrichment and depletion in soybean rhizosphere across all treatments, *LefSe* analysis between each pair of rhizosphere and soil samples were performed at the genus level. To improve the accuracy and robustness of the differential abundance analysis, any genus with a total count smaller than 50 was removed before LefSe analysis. Under one-against-all comparison mode, each genus with an α less than 0.05 and an LDA score greater than 2 was defined to be significantly different between rhizosphere and soil. Significantly enriched and depleted genera together with their LDA scores across treatments were merged to generate a tree file and an annotation file for GraphlAn visualization [71]. Any genus that was significantly enriched or depleted in the rhizosphere were annotated with red or blue colors respectively, while yellow color indicated no significant difference between rhizosphere and soil.

To infer the difference of microbe-microbe interaction patterns between soil types and among genotypes, samples were grouped based on treatments, i.e., Ag_Soil, Ag_WIL, Ag_DRT, Ag_CNR, Ag_NNW, Ag_SOJ, Ag_W82, For_Soil, For_WIL, For_DRT, For_CNR, For_NNW, For_SOJ and For_W82 (Ag for agricultural soil, For for forest soil, genotype abbreviations as defined in Table 2). To infer robust microbe-microbe interactions, any OTU with a total count smaller than 10 was removed to eliminate the confounding impacts introduced by these rare taxa. A co-occurrence correlation network between OTUs was calculated with *SparCC* algorithm with 20 interactions [72]. Corresponding *p*-values for each correlation were determined based on 200 iterations of the bootstrapping process. During the bootstrapping process, 200 sets of simulated count matrices were generated from the original count matrix. By comparing the SparCC correlation matrix generated using simulated datasets and that of the original dataset, p-values were calculated. For overall network topological traits comparison, each edge with a p-value less than 0.001 were kept for visualization. Further simplification of the networks was done by selecting the top 50 nodes with the largest connection degrees. The integrated network comprising all treatments was generated by uniting individual networks based on shared nodes, with different edge colors representing different treatments and different vertex colors depicting bacterial OTU (as defined in Fig. 8). The network visualization and topological properties measurements were done with the R package igraph [73].

To investigate the difference of potential ecological functions between bulk soil and rhizosphere microbiomes across all treatments, the R package *Tax4Fun* was used to predict microbial functional and metabolic capacities by linking 16S rRNA gene-based taxonomic profiles to pre-calculated KEGG references [74]. The predicted normalized KEGG pathway output was then used to investigate the enrichment of microbial pathways between soil and rhizosphere by DESeq2 [75]. Pathways with an adjusted *p*-value less than 0.01 and related to plant microbiome functions were selected for subsequent visualization in a heatmap using the *pheatmap* R package [76].

## Additional files

Additional file 1: **Figure S1** Sequence quality analysis using fastQC. **Figure S2** Sequencing depth distribution across all samples. **Figure S3** Rarefaction curve across all samples. **Figure S4** Genotype effects on soybean rhizosphere microbiome by comparing samples collected on the same date. **Figure S5** Correlation analysis between microbial Shannon diversities and network edge densities. **Figure S6** Integrated microbial global network including all significant correlations between OTUs. **Figure S7** Correlation analysis between network node degree and corresponding OTU relative abundance using both global network and Top50 network. **Figure S8** Individual microbial network constructed using top 50 nodes in terms of connection degree. (DOCX 15512 kb)

Additional file 2: Taxonomic annotation list for each node labelled in Fig. 3. (XLSX 53 kb)

Additional file 3: Bacterial genera and their abundance that specifically enriched in soybean rhizosphere when grown in one soil type but not the other. (XLSX 63 kb)

Additional file 4: Taxonomic annotation information for all OTUs that labelled in all network plots. (XLSX 294 kb)

## Data Availability

16S rRNA gene sequencing data and associated metadata were deposited to NCBI SRA repository under BioProject PRJNA474716. Detailed documentations of experiment design, sequencing process, statistical analysis and associated commands are available on Github (https://github.com/liufangbaishikele/Soybean_genotype_paper).

## Abbreviations

ANOVA: Analysis of variance
CAP: Constrained analysis of principal coordinates
KEGG: Kyoto encyclopedia of genes and genomes
LDA: Linear discriminant analysis
OTU: Operational taxonomic unit
PCoA: Principal coordinate analysis
PERMANOVA: Permutational multivariate analysis of variance
PGPR: Plant growth promoting rhizobacteria
