# Supplementary material for: Soil indigenous microbiome and plant genotypes cooperatively modify soybean rhizosphere microbiome assembly
Source: BMC Microbiol. 2019 Sep 2;19:201. doi: 10.1186/s12866-019-1572-x (PMC6720100; doi:10.1186/s12866-019-1572-x)

**Additional file 1: Supplementary figures**

**Soil indigenous microbiome and plant genotypes cooperatively modify soybean rhizosphere microbiome assembly**

Fang Liu^1^, Tarek Hewezi^3^, Sarah L. Lebeis^2^, Vince Pantalone^3^, Ernest C. Bernard^1^, Parwinder Grewal^4^, Margaret E. Staton^1*^

**Fig. S1 Sequence quality analysis using fastQC.**


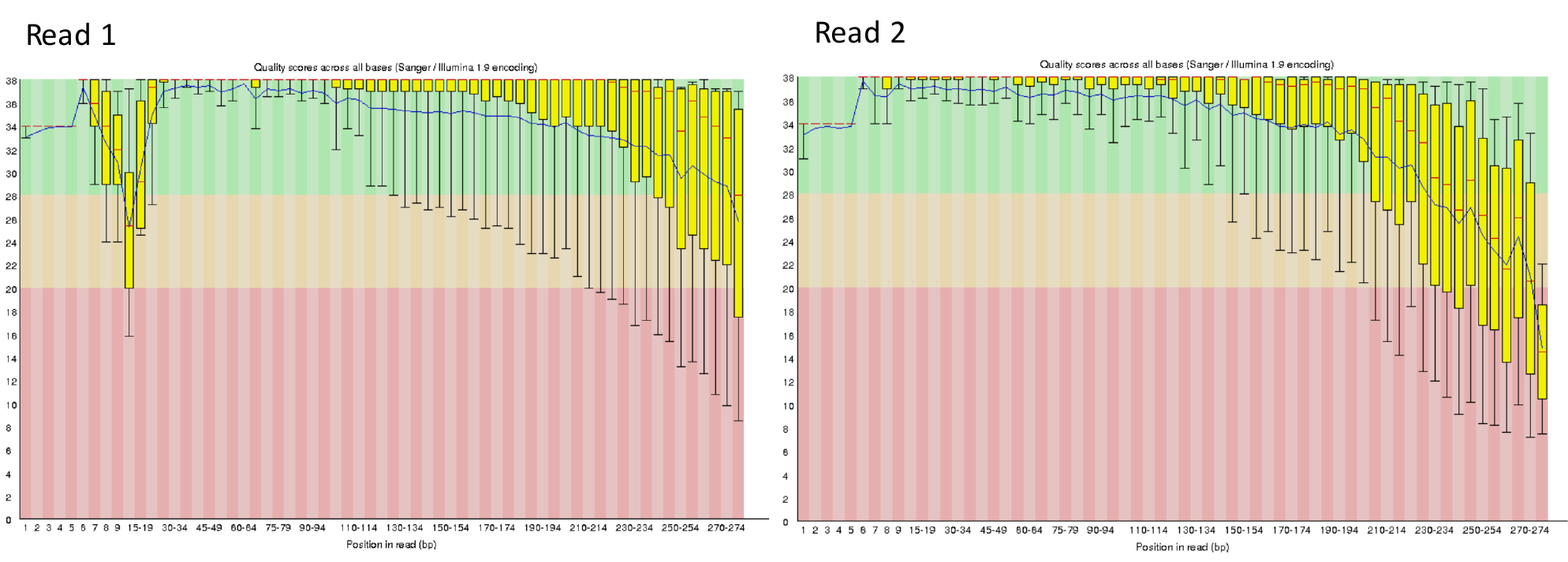


**Fig. S2 Sequencing depth distribution across all samples.**


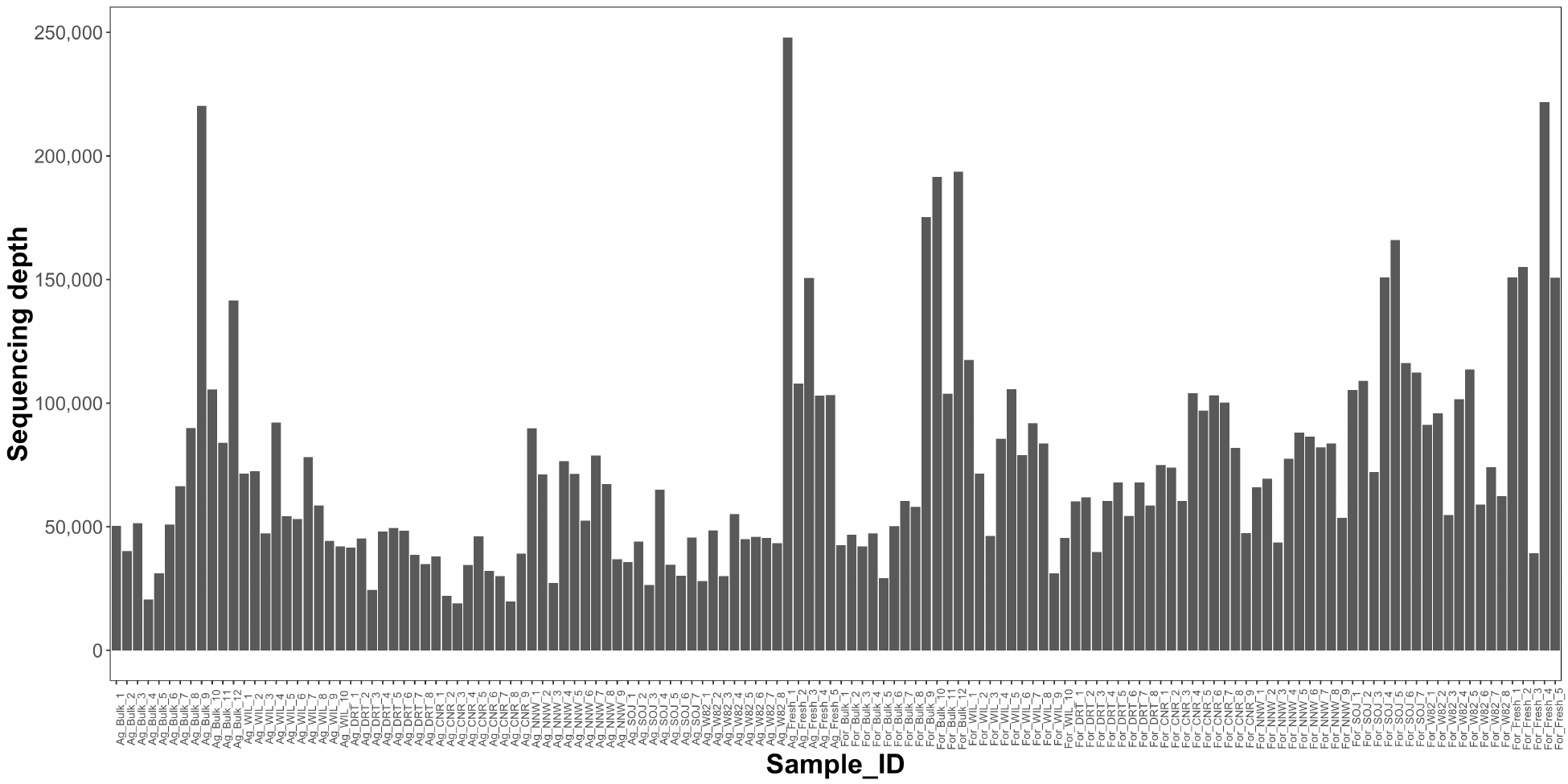


**Fig. S3 Rarefaction curve across all samples.**

The sample ID were labeled at the end of each line.


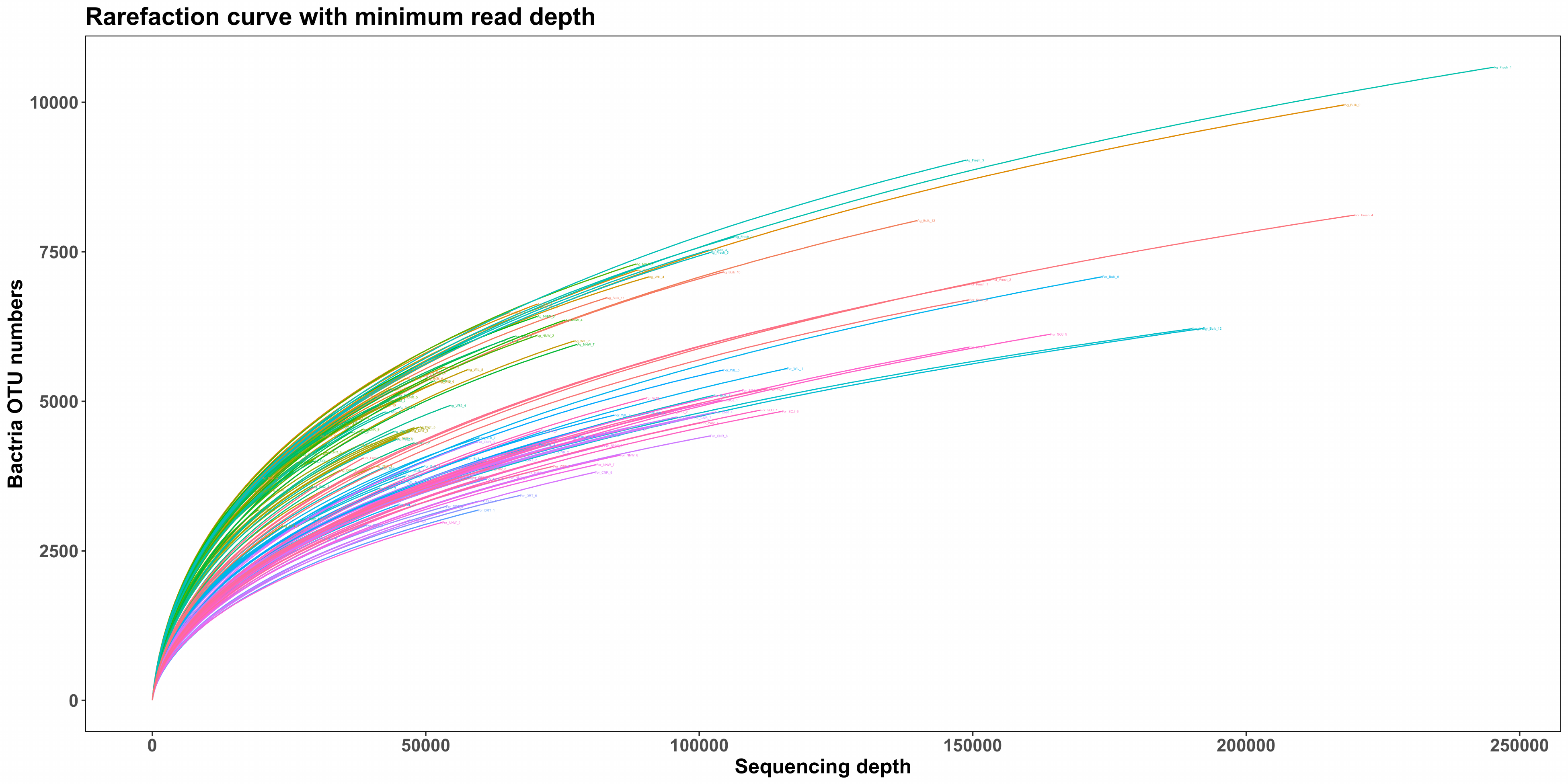


**Fig. S4 Genotype effects on soybean rhizosphere microbiome by comparing samples collected on the same date.**

To provide an example of pure genotype effect, rhizosphere samples belongs to William (WIL) vs non-nodulating William (NNW) mutant growing in agriculture soil that collected on 08-08-2016 and cyst nematode resitant (CNR) vs drought tolerant (DRT) collected on 08-26-2018 were compared and visualized using PCoA plot.


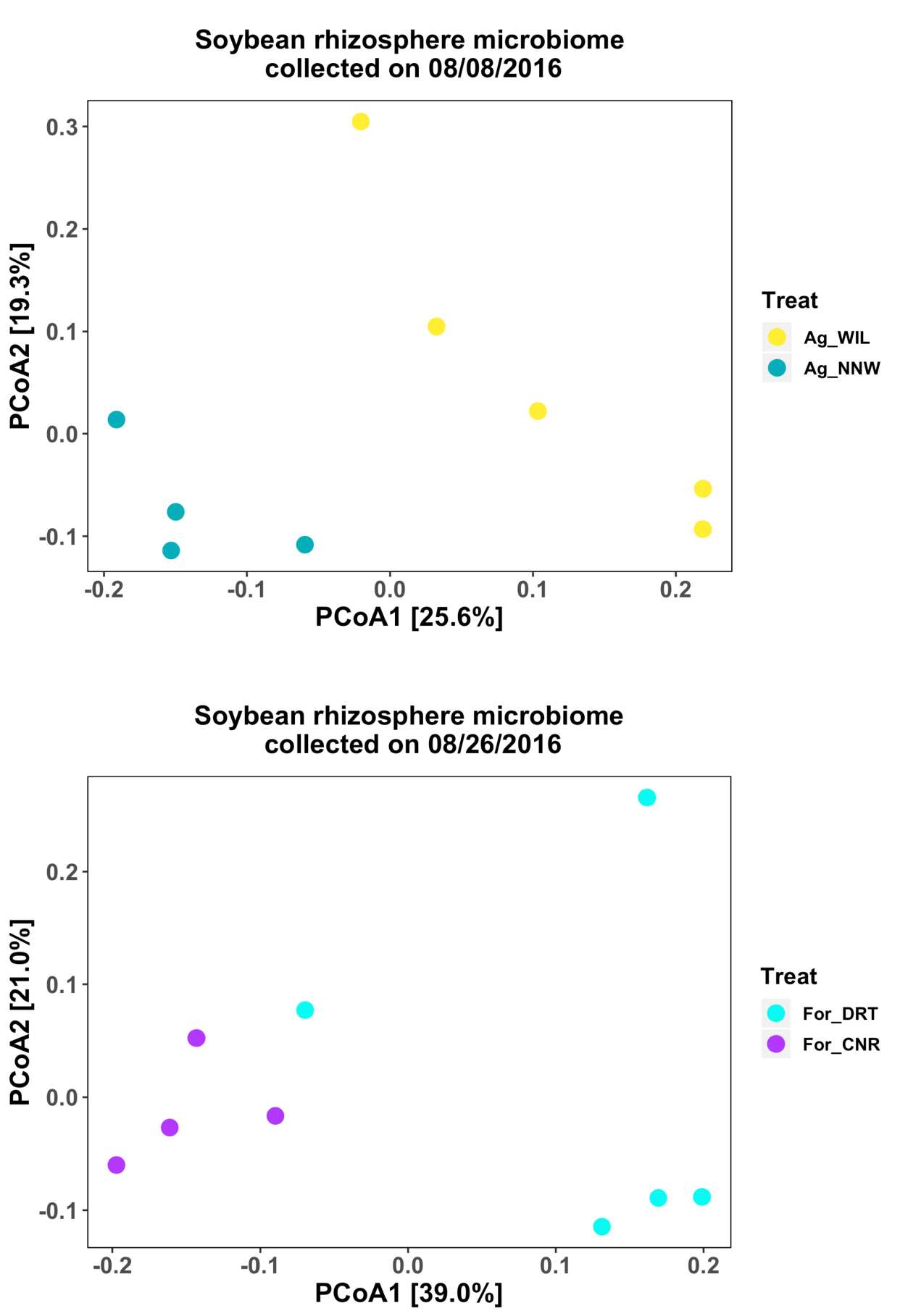


**Fig. S5 Correlation analysis between microbial Shannon diversities and network edge densities.**

The below correlation was calculated based on top50 network in which the first 50 nodes with the highest connections with other microbes were included.


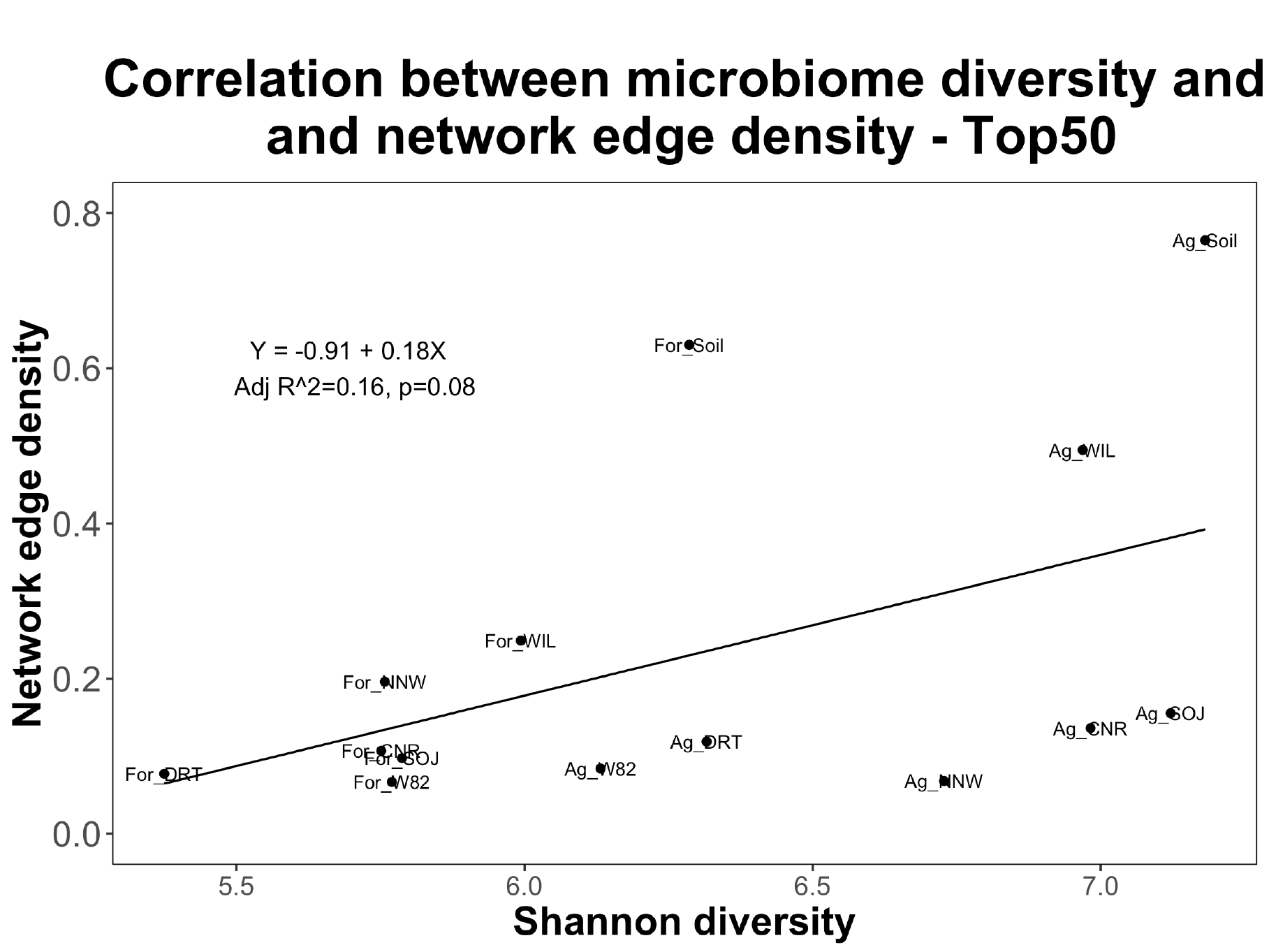


**Fig. S6 Integrated microbial global network including all significant correlations between OTUs.**

Inside of this network, each node represents one OTU and the correlations between OTUs were illustrated as edges. Nodes color were defined by their corresponding phylum, while edge color represents the treatment each edge belongs to. Whenever one edge was shared by several different treatments, the color was redefined by color mixer. Node size was represented by scaling the degree of connection of each node.


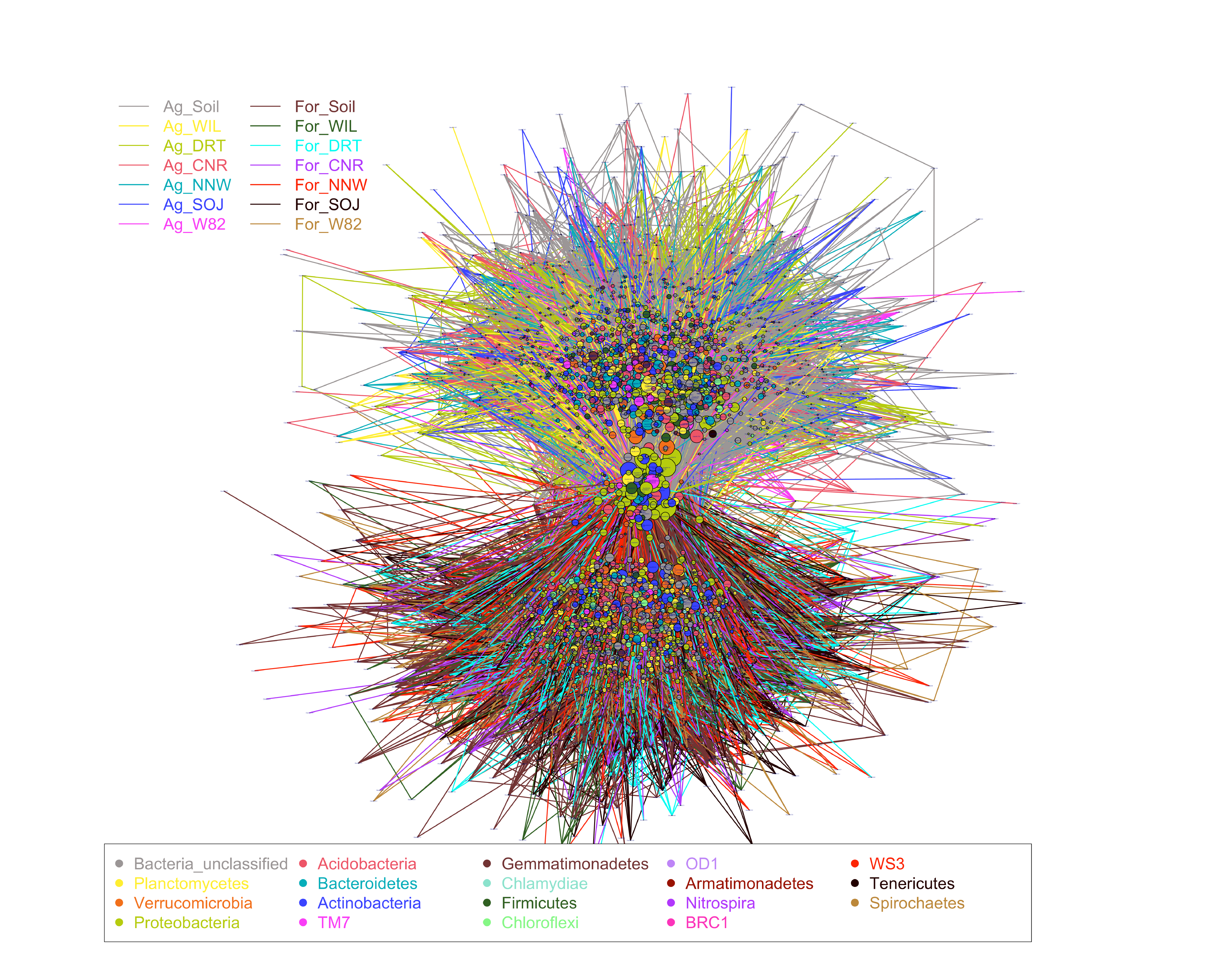


**Fig. S7 Correlation analysis between network node degree and corresponding OTU relative abundance using both global network and Top50 network.**

Each node was labeled with OTU_ID, whose taxonomy information could be found in Supplemental Table 3. Though p-value indicate significant correlation, only 10%-11% variance could be explained by this linear regression in both networks.


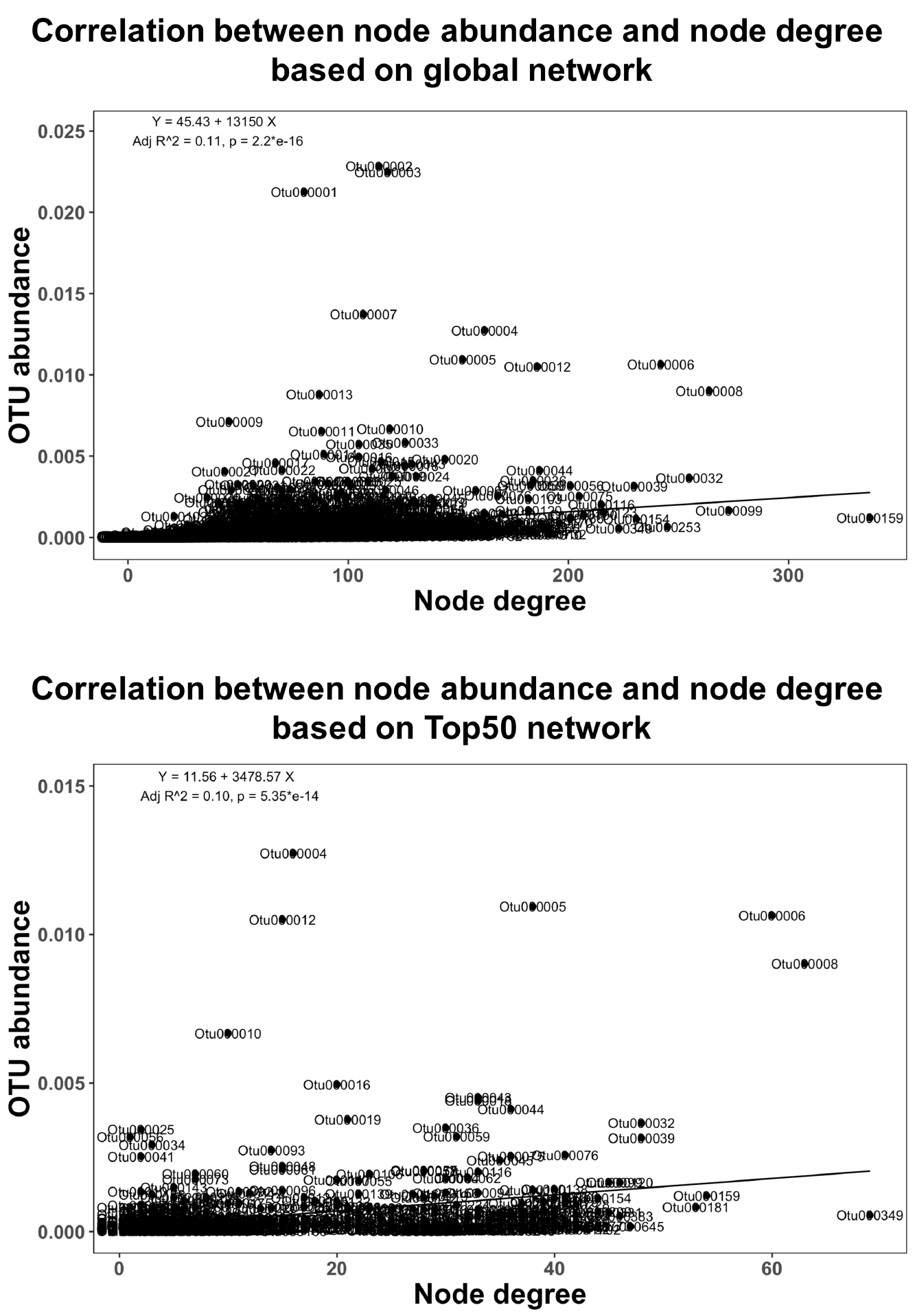


**Fig. S8 Individual microbial network constructed using top 50 nodes in terms of connection degree.**

In the below networks, nodes were colored based on phylum name as listed below. Red edge indicates negative correlation between OTUs while green edge represents positive correlation. Node size was defined based on connection degree. The bigger the node size, the more connections it has.


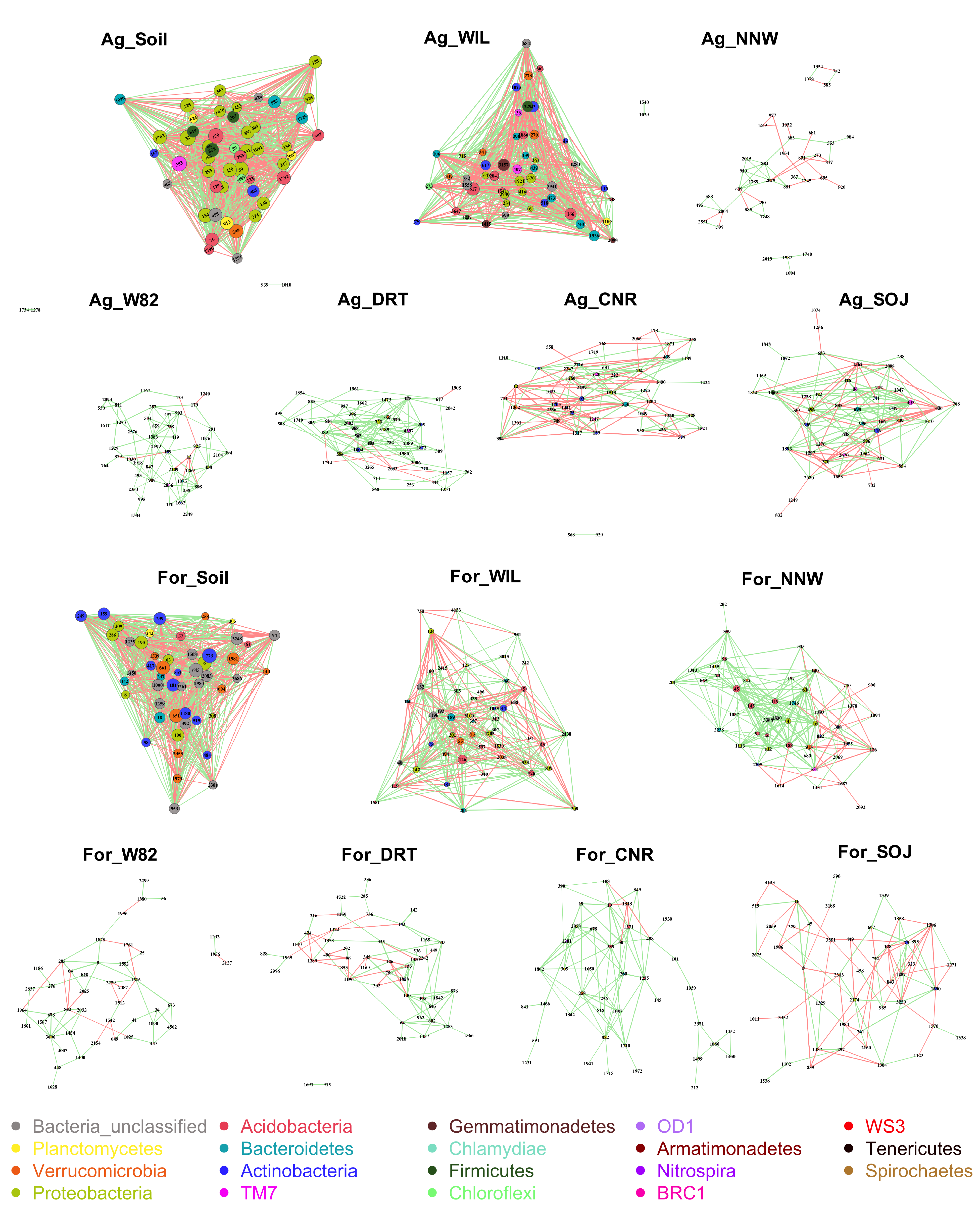

Supplement: Supplementary file 1 — Figure S1 Sequence quality analysis using fastQC. Figure S2 Sequencing depth distribution across all samples. Figure S3 Rarefaction curve across all samples. Figure S4 Genotype effects on soybean rhizosphere microbiome by comparing samples collected on the same date. Figure S5 Correlation analysis between microbial Shannon diversities and network edge densities. Figure S6 Integrated microbial global network including all significant correlations between OTUs. Figure S7 Correlation analysis between network node degree and corresponding OTU relative abundance using both global network and Top50 network. Figure S8 Individual microbial network constructed using top 50 nodes in terms of connection degree. (DOCX 15512 kb) [file 12866_2019_1572_MOESM1_ESM.docx]
